# The CYFIP2 Arg87Cys Mutation Reshapes the Interactome and Alters Stress Fiber-Associated Proteins

**DOI:** 10.1021/acsomega.5c12192

**Published:** 2026-10-12

**Authors:** Ísis V. Biembengut, Emanuella de C. Andreassa, Marlon D. M. dos Santos, Isabelle L. Z. Silva, Louise U. Kurt, Patrícia Shigunov, Paulo C. Carvalho, Tatiana A. C. B. de Souza

**Affiliations:** † Laboratory for Structural and Computational Proteomics, Instituto Carlos Chagas, Fundação Oswaldo Cruz (Fiocruz), Curitiba, Paraná 81350-010, Brazil; ‡ Stem Cell Basic Biology Laboratory, Instituto Carlos Chagas, Fundação Oswaldo Cruz (Fiocruz), Curitiba, Paraná 81350-010, Brazil

## Abstract

The Arg87Cys mutation in CYFIP2 has been implicated in early-onset developmental and epileptic encephalopathy (DEE), yet its molecular consequences remain incompletely understood. CYFIP2 functions as part of the WAVE regulatory complex (WRC), where it plays a key role in actin filament polymerization and interacts with RNA-binding proteins (RBPs) to regulate mRNA translation. Previous studies have demonstrated that this mutation alters the structure of CYFIP2, resulting in a gain of function in the WRC. Additionally, the mutation appears to promote the formation of intracellular granules that colocalize with RBPs; however, the impacts of this mutation on these interactions remain unclear. Here, we combined cross-linking mass spectrometry (XL-MS) and label-free quantitative proteomics to investigate how this mutation alters the protein interactome and actin regulatory pathways. XL-MS data revealed exclusive cross-links between actin and tropomyosin isoforms in the Arg87Cys variant, suggesting altered cytoskeletal dynamics. Quantitative proteomic analysis of patient-derived neural progenitor cells corroborated these findings by revealing an increase in the abundance of stress-fiber-associated proteins. Altogether, our results point to a broader impact of CYFIP2 mutations on cytoskeletal organization and protein interaction networks with potential implications beyond the nervous system. This is the first study to our knowledge to suggest a link between the Arg87Cys mutation and dysregulation of stress-fiber-related proteins, indicating possible disruption of other actin-processing pathways.

## Graphical abstract

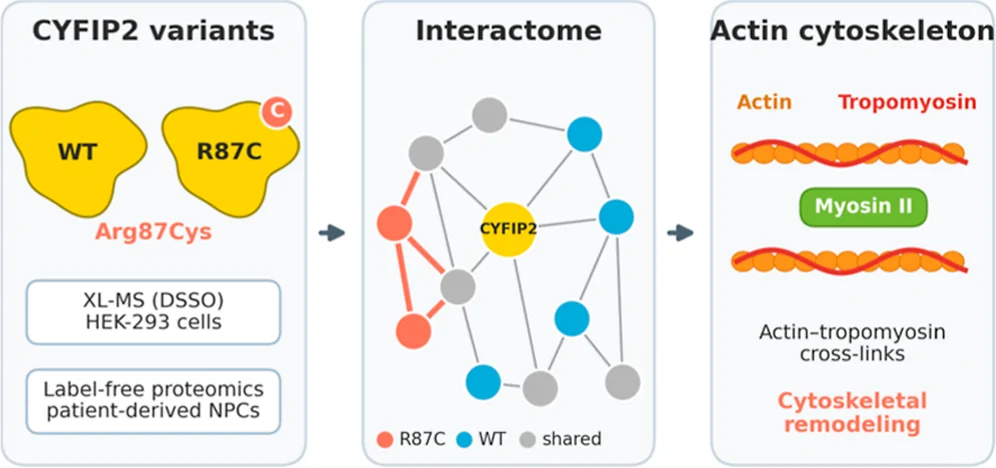

## Introduction

Developmental and epileptic encephalopathies (DEEs) comprise a rare group of neurodevelopmental syndromes characterized by impaired development arising from both the underlying cause, independent of epileptic activity, and the neurological dysfunction caused by epilepsy itself.^1^ The Arg87Cys mutation in the CYFIP2 protein has been identified in several patients with DEE, making it a focal point for studies aiming to elucidate the molecular underpinnings of this disease.^2,3^ Previous molecular dynamics studies have suggested that the Arg87Cys variant destabilizes CYFIP2–WAVE interactions and increases the flexibility of the 80–110 loop within the protein, supporting a mechanistic link to WAVE Regulatory Complex (WRC) dysregulation.^4^

CYFIP2 plays a pivotal role within the WRC, where it regulates actin filament polymerization.^5^ In addition, CYFIP2 interacts with fragile X mental retardation protein (FMRP), an RNA-binding protein (RBP), functioning as a regulator of mRNA translation.^6^ Protein–protein interactions are central to CYFIP2 function, particularly within the WRC, where the Arg87 residue forms a critical interface with WAVE1.^2,7^ Computational modeling has revealed structural shifts that may explain the gain-of-function observed in the mutant WRC.^4^ Although the Arg87Cys mutation has been shown to enhance WRC activity,^8,9^ its broader effects on the cellular protein interaction network and cytoskeletal regulation remain unclear.

Widely adopted approaches such as coimmunoprecipitation, used to investigate protein–protein interactions, are limited in their ability to distinguish direct from indirect contacts and to identify interaction sites.^10^ On the other hand, cross-linking mass spectrometry (XL-MS) overcomes these limitations. This technique employs chemical cross-linkers, molecules with a defined spacer arm length and reactive groups at both termini that covalently bind to specific functional groups (e.g., primary amines or thiols) on proximal proteins, enabling the subsequent identification of protein–protein interactions and contact sites via mass spectrometry.^11^

In this study, we employed XL-MS in HEK-293 cells transfected with either wild-type or Arg87Cys mutant CYFIP2, alongside label-free proteomics in patient-derived neural progenitor cells (NPCs), to investigate the effects of the mutation. This approach enabled us to map alterations in the protein interaction network and broader changes within the cellular interactome in the transfected HEK-293 system. This strategy was complemented by proteomic analyses of patient-derived cells, which revealed imbalances in stress-fiber-associated proteins, suggesting that the mutation may disrupt cytoskeletal regulation.

## Methods

### HEK-293 Cell Culture and Transfection

HEK-293 cells were cultured and transfected in triplicate with either wild-type (WT) or Arg87Cys mutant CYFIP2 plasmids using Lipofectamine 2000. Cells were maintained in DMEM with 10% fetal bovine serum at 37 °C and 5% CO_2_. After 24 h, cells were harvested, washed with phosphate-buffered saline (PBS), centrifuged, and the final pellets were stored at −80 °C for subsequent analysis.

### Sample Analysis Workflow

Protein–protein interactions were assessed through cross-linking mass spectrometry (XL-MS), following cross-linking, enzymatic digestion, SCX fractionation, and LC–MS/MS. To confirm expression, 20% of each pellet was lysed using freeze–thaw cycles, and the protein concentration was quantified using the Qubit assay. Western blot analysis was performed with antibodies against CYFIP2 and the HA-tag; GFP-transfected cells were used as a negative control. Protein bands confirmed successful expression of both WT and mutant CYFIP2 constructs. This workflow enabled high-confidence identification of interactome alterations between WT and Arg87Cys variants in a controlled cellular system.

### Western Blot

A small fraction (20%) of the total cell pellet from transfected cells was lysed to confirm CYFIP2 plasmid expression by Western blot analysis. The lysis was performed using three cycles of freeze–thaw using PBS buffer supplemented with a protease inhibitor cocktail. The protein concentration was measured using the Qubit Protein Assay (Invitrogen). Equal amounts of protein (10 μg) were separated by SDS-polyacrylamide gel electrophoresis and analyzed by Western blotting using rabbit polyclonal antibodies against CYFIP2 (ab95969, Abcam) and mouse monoclonal antibodies against the HA tag (26183, Thermo Fisher Scientific). As a negative control, lysate from HEK-293 cells transfected with pMax-GFP (Lonza Bioscience) was prepared using the same protocol.

### Transfected HEK-293 Cross-Linking

Cell pellets were resuspended in PBS to reach an approximate protein concentration of 4 mg/mL. Cross-linking was initiated by adding 2 mM disuccinimidyl sulfoxide (DSSO) diluted in dimethyl sulfoxide (DMSO), followed by a 15 min incubation at room temperature with agitation. The reaction was quenched by the addition of 20 mM Tris–HCl (pH 8.0), followed by an additional 15 min incubation at room temperature (25 °C) with agitation.

### Protein Digestion of Cross-Linked Whole Cells

Cross-linked cells were pelleted by centrifugation at 800*g* for 15 min, 4 °C, and subsequently resuspended in urea lysis buffer (8 M urea in 50 mM triethylammonium bicarbonate [TEAB], supplemented with a protease inhibitor cocktail; cOmplete, Roche). Cell lysis was performed by sonication in an ultrasonic bath for 10 min using alternating cycles of 30 s on/30 s off. Approximately 300 μg of cross-linked proteins was reduced with 5 mM dithiothreitol for 30 min at 56 °C, alkylated with 40 mM iodoacetamide for 25 min at room temperature, and digested sequentially with Lys-C endopeptidase (1:200 w/w) for 2 h and trypsin (1:100 w/w) overnight at 37 °C. Prior to trypsin digestion, samples were diluted with TEAB to reach a final urea concentration of 2 M. Digestion was stopped by the addition of 265 mM formic acid (FA), and peptides were desalted using Macro SpinColumns C18 cartridges (Harvard Apparatus). The resulting eluates were quantified using Qubit Protein Assay (Invitrogen).

### SCX Fractionation

Each digested peptide sample from the total cell extract was loaded onto a Capto HiRes S 5/50 column (Cytiva) using an ÄKTA UPC 100 system and separated over a 72-column-volumes (CV) gradient. Fractions were collected throughout the gradient at intervals of 2 mL. Based on the chromatogram profile of each sample, fractions corresponding to the 25 to 65 mL elution range were selected and pooled to yield eight final fractions per sample. The pooled fractions were desalted using C8 StageTips.

### LC–MS/MS Data Acquisition of Cross-Linked Samples

Samples were separated by reverse-phase high-performance liquid chromatography (HPLC)­using a nanochromatography system, UltiMate 3000 (Thermo Fisher Scientific Inc.). Separation was performed on in-house packed reverse-phase columns containing ReprosilPur C18 stationary phase (3 μm diameter beads and 120 Å pore size; Dr. Maisch), operated at a flow rate of 250 nL/min over a 127 min gradient. The system was coupled to an Orbitrap Fusion Lumos mass spectrometer (Thermo Fisher Scientific) located at the Proteomics and Mass Spectrometry facility at the Carlos Chagas Institute, Fiocruz-PR. MS1 scans were acquired in an Orbitrap with the *m*/*z* range of 375–1600, a resolution of 120,000, standard AGC target, maximum injection time of 50 ms, and 60 s dynamic exclusion. MS2 scans were also acquired in an Orbitrap at a mass resolution of 60,000, with a maximum injection time of 118 ms and an isolation window of 1.6 *m*/*z*. Precursor ion charge states for MS2 were set from 4+ to 8+, and peptide fragmentation was performed using stepped collision energy of 21%, 27%, and 33%. Data acquisition was performed with a 2 s cycle time. Scan functions and solvent gradients in the nanoliquid chromatography (nLC) were controlled by Xcalibur 4 software (Thermo Fisher Scientific).

### Culture and Lysis of H1 and SP1C8-Derived NPCs

NPCs derived from a patient carrying the CYFIP2 Arg87Cys mutation (C8) and from H1 human embryonic stem cells (H1) were cultured as previously described Silva et al. (2023). Protein extraction was carried out using 0.1% RapiGest in combination with 1.0 mm zirconia bead agitation. After quantification via Qubit fluorimetric assay, 100 μg of protein per sample was reduced with 10 mM dithiothreitol for 30 min at 60 °C, alkylated with 30 mM iodoacetamide for 25 min in the dark, and digested overnight with trypsin (1:50 w/w) at 37 °C. Digestion was stopped with 265 mM FA, followed by a 40 min incubation to degrade RapiGest. Samples were centrifuged to remove insoluble material and desalted using C8 StageTips.

### LC–MS/MS Data Acquisition of H1 and SP1C8-Derived Neural Progenitor Samples

Samples were separated by reverse-phase high-performance liquid chromatography (HPLC) using a nanochromatography system, UltiMate 3000 (Thermo Fisher Scientific Inc.). Separation was performed on in-house packed reverse-phase columns containing ReprosilPur C18 stationary phase (3 μm diameter beads and 120 Å pore size; Dr. Maisch), operated at a flow rate of 300 nL/min over a 127 min gradient. The system was coupled to an Orbitrap Fusion Lumos mass spectrometer (Thermo Fisher Scientific) located at the Proteomics and Mass Spectrometry Facility of the Carlos Chagas Institute (Fiocruz-PR). MS1 scans were acquired in the Orbitrap with a mass-to-charge (*m*/*z*) range of 300–1500, at a resolution of 60,000, using a standard AGC target, maximum injection time of 50 ms, and dynamic exclusion set to 60 s. MS2 scans were acquired in the Orbitrap at a resolution of 15,000, with a maximum injection time of 22 ms and an isolation window of 1.6 *m*/*z*. Precursor ion charge states for MS2 were set from 2+ to 7+, and peptide fragmentation was performed using a fixed collision energy of 30%. Data acquisition was conducted using a 2 s cycle time. Scan functions and solvent gradients in the nano-LC system were controlled by Xcalibur 4 software (Thermo Fisher Scientific).

### Proteomics Analysis

Chromatogram analysis and run quality control were performed using Raw Vegetable 2.0.^12^ Peptide identification and label-free quantification were conducted using PatternLab for Proteomics V (version 5.0).^13^ Protein sequences for *Homo sapiens* were retrieved from Swiss-Prot (May 2023). Because of the high sequence similarity between CYFIP1 and CYFIP2, CYFIP1 sequences were excluded from the database search to minimize ambiguous peptide assignment arising from shared tryptic peptides. CYFIP2 plasmid sequences were included, and the identification of CYFIP2-specific peptides confirmed the detection of the transfected protein in the analyzed samples. A target-decoy database was constructed by generating reversed versions of each sequence and incorporating common mass-spectrometry contaminants. Mass spectra were searched using the Comet 2021.01 rev. 0 search engine with a peptide mass tolerance of 35 ppm, precursor mass range from 500 to 6000 Da, up to two missed cleavages, a precursor mass tolerance of 40 ppm, and a fragment bin tolerance of 0.02. Carbamidomethylation of cysteine was set as a fixed modification, and oxidation of methionine was set as a variable modification. Peptide identifications were grouped based on the charge state and enzyme specificity. A false-discovery rate (FDR) threshold of 2% at the peptide level was applied, determined by the number of decoy hits. Additional filters required a minimum sequence length of six amino acids and a protein score of above 2.1. Finally, identifications with mass deviations exceeding 10 ppm from theoretical values were excluded, resulting in a final protein level FDR below 1% across all search results.

Relative quantification was based on extracted ion chromatograms (XICs) from two biological replicates per condition, each analyzed in duplicate as a technical replicate. The mass accuracy tolerance for precursor ions was set to 10 ppm, and MS1 tolerance was set to 1. Only proteins with at least one uniquely quantified peptide were retained for the downstream analysis. For the comparison between C8 and H1 samples, a TFold analysis was performed with a BenjaminiHochberg (BH) correction using a *q*-value threshold of 0.05.

### Cross-Linking Mass Spectrometry Analysis

Chromatogram analysis and run quality checks were performed using Raw Vegetable 2.0.^12^ Cross-linked peptides, peptide pairs, and protein–protein interactions (PPIs) were identified using Scout (version 1.5.1)^14^ and quantified using Quin 2.0.^15^ Each biological replicate was analyzed independently by using a refined database generated from a proteomic search. The search parameters were set to a mass tolerance of 10 ppm for MS1 and 20 ppm for MS2 with a precursor mass range of 500 to 6000 Da. Up to three missed cleavages were allowed, and peptides were required to have a minimum length of six amino acids. Carbamidomethylation of cysteine was set as a fixed modification, and methionine oxidation was treated as a variable modification. Postprocessing steps applied an FDR threshold of 5% at the levels of cross-linked spectral matches (CSMs), residue pairs, and PPIs.

### Data Analysis

Python 3.13 scripts were developed to filter the data, generate graphs, and enhance data visualization. In addition, SRplot^16^ was used for graph construction and data visualization. Gene Ontology (GO) analyses were performed using GOATOOLS,^17^ employing a *H. sapiens* background gene list and focusing on differentially abundant proteins identified in the comparison between C8 and H1 NPCs. Protein interaction networks were constructed using the STRING database^18^ by querying all proteins identified in the cross-linking analysis at the cross-linked spectral match (CSM) level, along with a CYFIP2-centered subnetwork filtered by a confidence cutoff score of 0.4. Network visualizations were generated using Cytoscape (v.3.10.3) (https://cytoscape.org). To simplify the representation, orphan nodes, defined as proteins with no proposed interactions, were excluded from the final network graphics. Quantitative comparisons were performed across three biological replicates per condition. Given the limited number of peptide pairs meeting the filtering criteria (*p* < 0.05 and ≥2-fold change in abundance between conditions), no correction for multiple testing was applied; this represents a methodological limitation of the present analysis.

## Results

We performed cross-linking mass spectrometry (XL-MS) in three biological replicates of HEK-293 cells transfected with either wild-type (CYFIP2 WT) or Arg87Cys constructs. The transfection efficiency was confirmed by Western blot (Figure S1). Across all experiments, we identified a total of 7765 CSMs at a 5% FDR, corresponding to 4253 residue pairs and 714 protein–protein interactions (PPIs) (Tables S1–S3, Figures S2 and S3). In the CYFIP2 WT condition, 4692 CSMs from 746 proteins were identified, while the Arg87Cys condition yielded 3073 CSMs from 560 proteins. Approximately 60% of the cross-linked proteins were common to both conditions. However, 258 proteins were uniquely detected in the WT samples, and 72 proteins were exclusive to the Arg87Cys samples (Figure 1).

**Figure 1:**
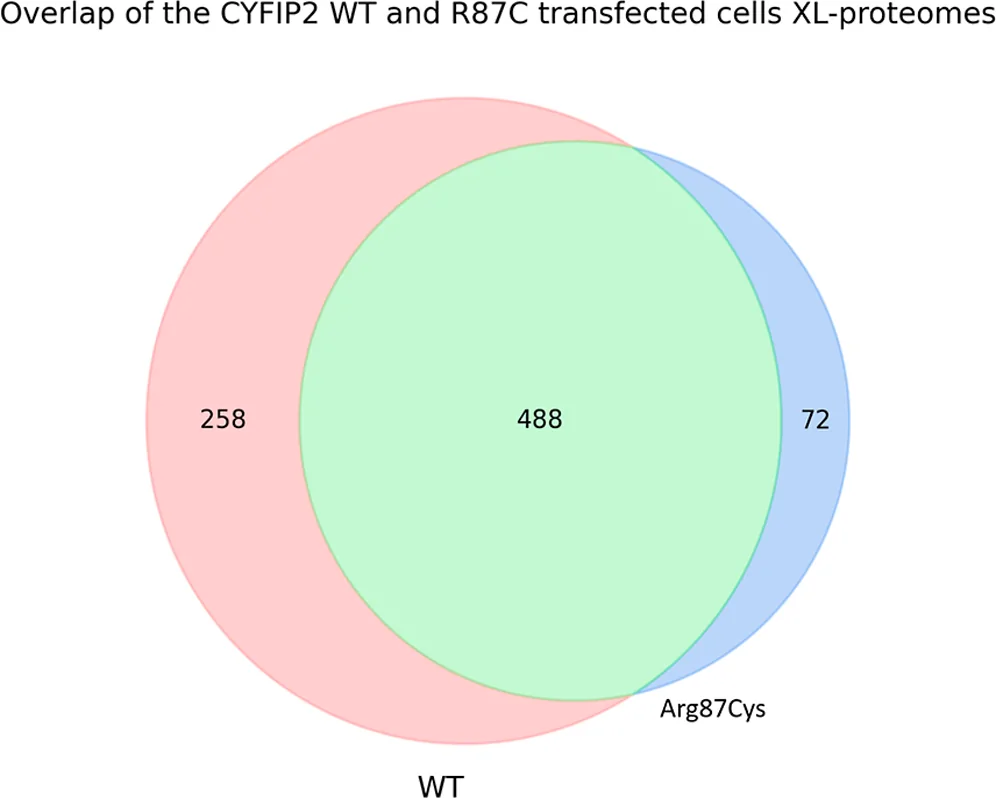
Venn diagram showing the overlap of cross-linked proteomes (XL-proteomes) from HEK-293 cells transfected with CYFIP2 WT and Arg87Cys. Among the 818 total proteins identified, 488 were shared between both conditions, while 258 and 72 were unique to the WT and Arg87Cys conditions, respectively.

Quantitative analysis was done at the peptide pairs level, by grouping XIC values of individual CSMs among replicates. Having these values at hand, fold changes and *p*-values (by Student’s *t*-test) were then calculated, so that those peptide pairs with a *p*-value lower than 0.05 and a quantification difference of at least 2× between the WT and Arg87Cys conditions would be considered differentially abundant. Few peptide pairs met these criteria, and no statistically significant global differences were observed between the WT and Arg87Cys conditions (Table S4). However, notable qualitative differences in interaction specificity were observed. Table 1 summarizes cross-links uniquely identified in at least two of the three biological replicates for each condition. In WT cells, heteromeric cross-links were detected among ATP synthase subunits (ATP5F1C–ATP5F1E, ATP5F1D–ATP5F1E, ATP5PF–ATP5PD), mitochondrial complex I components (NDUFA5–NDUFA7), and structural or signaling proteins such as YWHAQ and ISCA1. In contrast, Arg87Cys samples exhibited exclusive cross-links between actin isoforms (ACTG2, ACTA1, ACTB) and tropomyosins (TPM2, TPM3), suggesting mutation-induced remodeling of cytoskeletal architecture.

**Table 1:** CSMs Detected Exclusively in WT or Arg87Cys Conditions, Identified in at Least Two Biological Replicates. Functional Annotations Were Obtained from UniProt and GO Terms

protein 1	protein 2	condition	number of replicates	functional annotation
ACAT1	ACAT1	WT	3	mitochondrial thiolase; fatty acid metabolism
ATP5F1C	ATP5F1E	WT	2	ATP synthase subunits; oxidative phosphorylation
ATP5F1D	ATP5F1E	WT	3	ATP synthase subunits; mitochondrial energy production
ATP5PF	ATP5PD	WT	3	ATP synthase peripheral stalk components
ATP5PF	ATP5PB	WT	3	ATP synthase peripheral stalk and membrane subunits
LDHA	LDHB	WT	2	lactate dehydrogenase A/B; anaerobic glycolysis
LDLR	VIM	WT	2	LDL receptor and vimentin; lipid uptake and cytoskeletal structure
MAGOHB/MAGOH	RNPS1	WT	2	spliceosome components; mRNA processing
NDUFA7	NDUFA5	WT	2	complex I (NADH/ubiquinone oxidoreductase); mitochondrial respiration
RPS2	RPS9	WT	2	ribosomal proteins; translation
SOD1	SOD1	WT	2	superoxide dismutase; oxidative stress response
YWHAQ	ISCA1	WT	2	signal transduction and Fe–S cluster assembly
ACTG/ACTA/ACTB	TPM2/TPM3	Arg87Cys	2	actins and tropomyosins; cytoskeletal organization
MRPL38	MRPL52	Arg87Cys	2	mitochondrial ribosomal proteins; protein synthesis in mitochondria
RPS24	RPS9	Arg87Cys	2	ribosomal proteins; translation

To contextualize these findings, we mapped the proteins identified by XL-MS, alongside known CYFIP2 interactors, using the STRING database with a confidence threshold of 0.4 (Figure 2). In the resulting network, red nodes represent proteins uniquely identified in the Arg87Cys condition, blue nodes are exclusive to WT, and gray nodes are shared between both conditions. Notably, actin–tropomyosin interactions appeared exclusively in Arg87Cys samples, whereas WT exhibited predominantly mitochondrial-associated interactions. Because experimentally resolved structures and high-confidence predicted models are not currently available for the mitochondrial protein assemblies cross-linked in this data set, Cα–Cα Euclidean-distance validation against the theoretical cross-linker reach (∼30 Å for DSSO) could not be performed. The differential mitochondrial cross-links reported here should therefore be interpreted as indicative of spatial proximity within mitochondrial microenvironments rather than as definitive evidence of direct binary protein–protein interactions. Targeted structural studies, including cryo-EM of the relevant subcomplexes and orthogonal biochemical validation, will be required in future work to formally discriminate direct contacts from indirect proximity.

**Figure 2:**
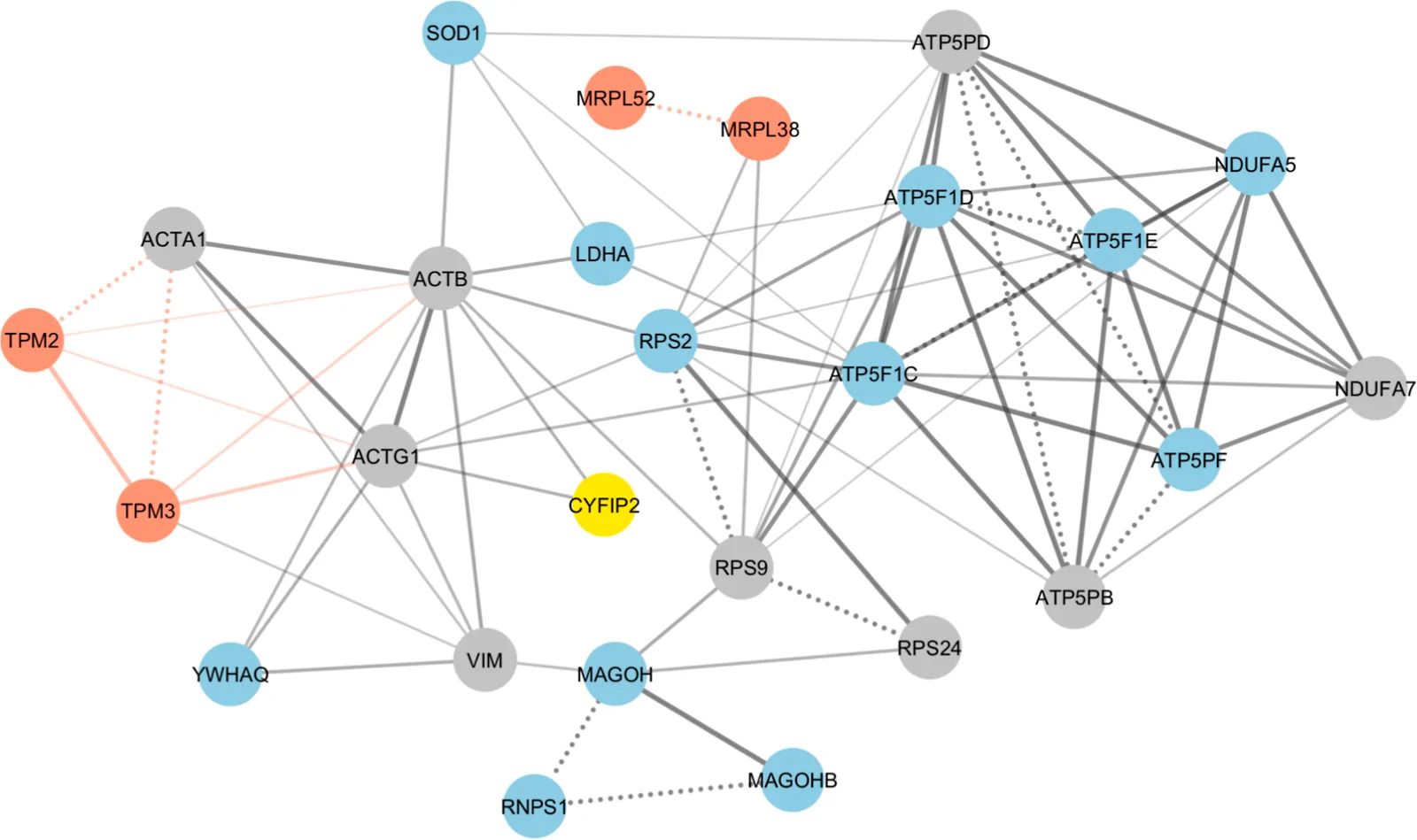
STRING-based protein interaction network showing CYFIP2-centered interactions. Red nodes represent proteins whose interlinks were identified exclusively in the Arg87Cys condition, while blue nodes correspond to those found only in WT cells. Gray nodes indicate proteins whose interlinks were detected in both conditions. CYFIP2 is highlighted in yellow. Highlighted edges (in bold) indicate XLs detected exclusively in the Arg87Cys condition across two biological replicates. Dotted edges represent CSMs found within our data set.

The absence of key mitochondrial protein–protein interactions (PPIs) in the Arg87Cys condition may indicate alterations in energy metabolism. Nonetheless, the detection of alternative ATP synthase subunit interactions (e.g., ATP5F1A–ATP5F1B) suggests that mitochondrial activity is at least partially preserved. Although these observations raise the possibility of altered mitochondrial function in the Arg87Cys condition, this hypothesis would require confirmation through orthogonal functional assays, such as measurements of cellular respiration or ATP levels. In parallel, the exclusive detection of actin–tropomyosin cross-links in Arg87Cys samples may reflect mutation-driven cytoskeletal remodeling. These interactions, absent in WT, could underlie changes in intracellular transport, cell morphology, or synaptic plasticity, processes that are particularly critical in neuronal contexts.

To further investigate the impact of the Arg87Cys mutation, we analyzed the proteome of NPCs derived from a patient carrying the mutation (C8) and compared it to NPCs derived from wild-type H1 human embryonic stem cells. A total of 192 proteins were identified as differentially abundant, with 102 upregulated in C8 cells and 90 in H1 cells (Table S5, Figure S4). As illustrated in the volcano plot (Figure 3A), several stress fiber-associated proteins, including CALD1, TRIP6, LIMA1, TLN1, and PALLD, were significantly upregulated under the mutant condition. GO enrichment analysis of these differentially abundant proteins (DAPs) highlighted terms related to cytoskeletal regulation, including “stress fiber” and “focal adhesion”, as well as chromatin-associated terms such as “chromatin looping”, “cohesin loader activity” and “chromatin extrusion motor activity” (Figure 3B). These findings suggest that the Arg87Cys mutation alters both cytoskeletal organization and chromatin regulatory pathways in NPCs.

**Figure 3:**
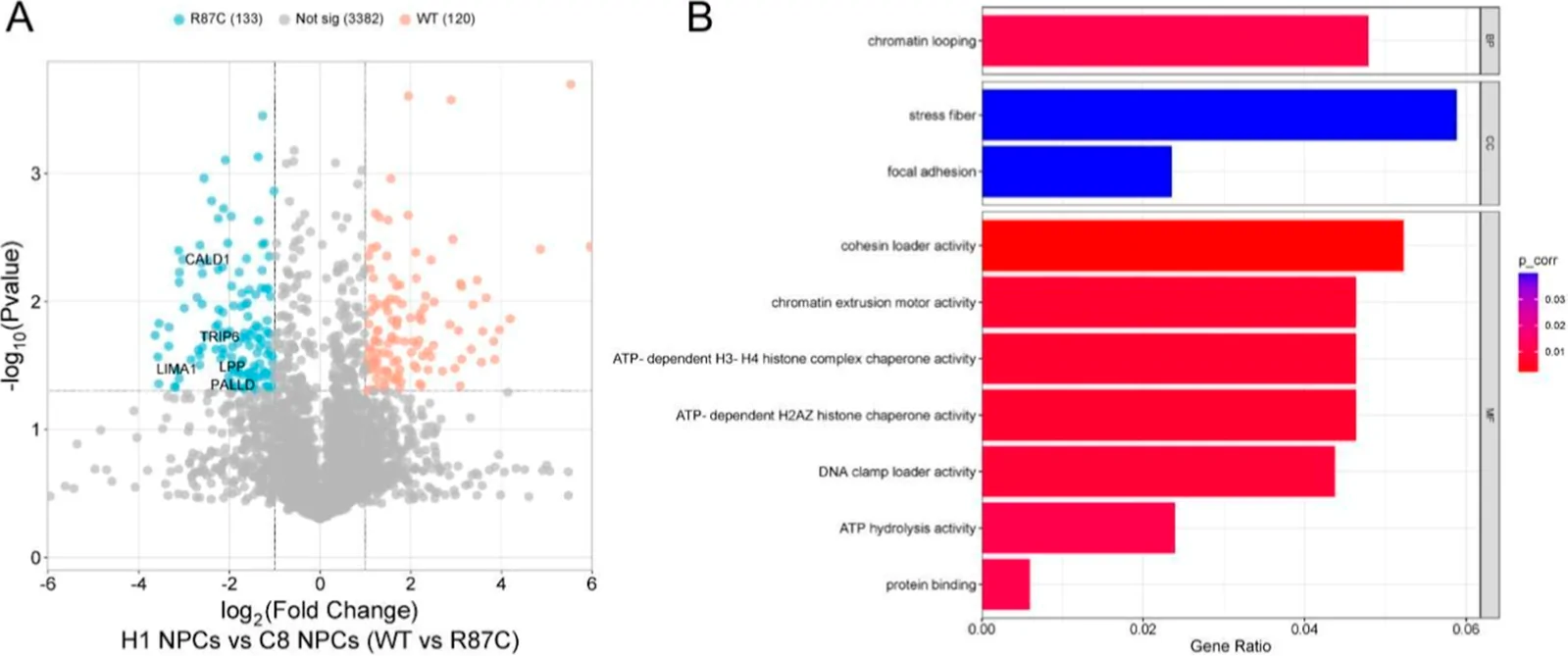
DAPs in H1 (WT) vs C8 (Arg87Cys) NPCs. (A) Volcano plot showing proteins with significant changes in abundance (*p* < 0.05, fold change >2.0). Highlighted proteins are associated with stress fiber formation. (B) GO enrichment analysis of DAPs, categorized by biological process, cellular component, and molecular function.

The enrichment of stress fiber-associated proteins in C8 cells aligns with the XL-MS data, which revealed increased actin–tropomyosin interactions under the mutant condition. Together, these findings support a model in which the CYFIP2 Arg87Cys mutation reshapes the cellular interactome toward cytoskeletal components, particularly those linked to stress fiber assembly. Given CYFIP2’s established role in actin regulation via the WRC, the mutation likely enhances actin polymerization dynamics, leading to broader changes in cellular architecture. While such alterations are well recognized in neurons, they may also have functional consequences in non-neuronal cells involved in mechanotransduction.

Despite the compelling convergence of proteomic and interactomic evidence, certain limitations must be acknowledged. Interactions exclusive to the Arg87Cys condition may also occur in WT cells at a lower abundance, potentially falling below detection thresholds. Future studies employing complementary techniques such as coimmunoprecipitation and proximity labeling will be crucial to validate these associations. Nonetheless, our findings highlight the potential role of cytoskeletal dysregulation in the pathophysiology associated with the CYFIP2 Arg87Cys variant.

## Discussion

Our comparative cross-linking mass spectrometry (XL-MS) analysis revealed distinct interactome profiles between HEK-293 cells expressing wild-type CYFIP2 and those expressing the Arg87Cys variant. In the WT condition, we identified heteromeric interlinks among ATP synthase subunits (e.g., ATP5F1C–ATP5F1E, ATP5F1D–ATP5F1E, ATP5PF–ATP5PD, ATP5PF–ATP5PB) as well as between mitochondrial Complex I proteins (NDUFA5–NDUFA7). These interactions are consistent with active oxidative phosphorylation and support the presence of intact mitochondrial function in WT cells.

By contrast, these specific CSMs were absent under the Arg87Cys condition. This absence may suggest mitochondrial dysfunction or a potential metabolic shift toward glycolysis, a hallmark of cellular stress, commonly referred to as the Warburg effect (Warburg, 1956). However, this interpretation warrants caution. Notably, we also detected heteromeric links involving other ATP synthase subunits in the Arg87Cys variant (e.g., ATP5F1A–ATP5PD, ATP5F1A–ATP5F1B), suggesting that mitochondrial metabolism is not entirely suppressed, but may instead be altered in composition or regulatory dynamics.

Interestingly, we detected an exclusive heteromeric interaction between actins (ACTG2, ACTG1, ACTA2, ACTA1, ACTB, ACTBL2) and tropomyosins (TPM2, TPM3) under the Arg87Cys condition. No such actin–tropomyosin cross-links were observed in the WT data set, despite the presence of both protein families under both conditions. This selective interaction may reflect a reorganization of cytoskeletal structures triggered by the Arg87Cys variant (Figure 4). However, differences in cross-link detection may also reflect mutation-associated conformational changes that alter residue accessibility to the cross-linking reagent, rather than solely representing strengthened or novel physical interactions. These findings align with previous molecular dynamics simulations, which showed that the Arg87Cys mutation increases the flexibility of the 80–110 loop and weakens key intra- and intermolecular interactions between CYFIP2 and WAVE1, potentially destabilizing the WRC.^4^ Although previous molecular dynamics simulations suggested that the Arg87Cys substitution does not create favorable spatial conditions for intramolecular disulfide bond formation within CYFIP2, the possibility that the introduced cysteine residue may influence intermolecular interactions or redox-sensitive protein behavior cannot be excluded. Future studies employing nonreducing biochemical assays and structural approaches will be important to investigate these possibilities.

**Figure 4:**
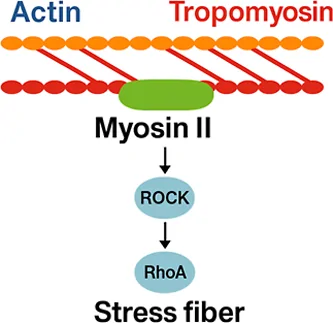
Schematic representation of stress fiber assembly. Stress fibers are actomyosin structures formed by parallel actin filaments (orange) cross-linked by tropomyosins (red), which stabilize and regulate the filament network. Myosin II (green) binds to these filaments to generate contractile force. This assembly is regulated by the RhoA/ROCK signaling pathway, which promotes actin polymerization and myosin II activation. In the context of the Arg87Cys mutation, the exclusive actin–tropomyosin cross-links identified in our XL-MS analysis suggest a shift toward stress fiber formation, potentially affecting cellular tension, morphology, and migratory capacity.

Actin isoforms are highly conserved proteins essential for cytoskeletal integrity and dynamic remodeling, particularly in neurons and muscle cells.^19^ Tropomyosins, encoded by TPM genes, stabilize actin filaments and are critical for the formation of specialized cytoskeletal structures such as stress fibers.^20,21^ Although CYFIP2 is not known to directly interact with TPM proteins, its well-established role in actin polymerization through the WRC^5,22^ may indirectly influence TPM–actin interactions and cytoskeletal organization.

The Arg87Cys mutation in CYFIP2 has been previously associated with impaired actin dynamics and altered neuronal morphology.^9,23^ The appearance of actin–tropomyosin CSMs exclusively in the Arg87Cys condition may therefore reflect compensatory remodeling or maladaptive cytoskeletal restructuring. Although no current evidence directly links CYFIP2 to the regulation of TPM2 or TPM3 in the context of epilepsy, our findings suggest a potential indirect connection mediated by altered actin dynamics.

A key limitation of our XL-MS data set is the depth of coverage. Since actin–tropomyosin interactions were detected exclusively in the Arg87Cys condition, we cannot rule out the possibility that these interactions also occur in WT cells at lower abundance but remained below the detection threshold. Nonetheless, the identification of multiple actin isoforms and TPM proteins in both data sets lends support to the biological relevance of this finding. Future studies combining coimmunoprecipitation^10^ with cross-linking mass spectrometry will be essential to validate the interactions and determine whether they are upregulated or uniquely enriched in Arg87Cys cells.

Further supporting our XL-MS findings, label-free proteomics of NPCs derived from induced pluripotent stem cells of a patient carrying the CYFIP2 Arg87Cys mutation revealed 192 DAPs when compared to NPCs derived from H1 embryonic stem cells. GO enrichment analysis^17^ identified “protein binding” as one of the most significantly enriched terms, likely reflecting the upregulation of actin-binding proteins and components of the WRC.^5^

Notably, the “stress fiber” category also emerged as significantly enriched and included CALD1, TRIP6, PALLD, LIMA1, and LPP, proteins associated with actin filament stabilization and cytoskeletal remodeling.^24–27^ This suggests a broader disruption of cytoskeletal dynamics that extends beyond WRC-mediated actin branching. However, because the NPC proteomic analyses reflect steady-state profiles obtained after prolonged cell culture and propagation, the observed abundance changes may represent a combination of direct mutation-associated effects and longer-term compensatory cellular adaptations to cytoskeletal stress. The convergence of stress fiber components in both interactome and proteome data sets reinforces the hypothesis that cytoskeletal dysregulation represents a core pathomechanism in CYFIP2-associated neurodevelopmental disorders. The XL-MS and label-free proteomics analyses were performed in different cellular models, namely HEK-293 cells and NPCs, respectively. Because these systems differ substantially in cellular identity, protein expression profiles, and physiological context, the interaction networks and abundance changes identified are not directly comparable at the molecular level. Therefore, the observed convergence of cytoskeleton-related alterations across both data sets should be interpreted as supportive rather than definitive evidence of a shared pathogenic mechanism associated with the Arg87Cys variant.

Taken together, our findings suggest that the CYFIP2 Arg87Cys mutation induces selective remodeling of mitochondrial and cytoskeletal networks, with particular impact on actin–tropomyosin interactions and stress fiber-associated proteins. Given the central role of actin dynamics in neurodevelopment and synaptic plasticity,^28^ such alterations may underlie key phenotypic features observed in CYFIP2-related epileptic encephalopathies.^1,2,29^ Future studies integrating quantitative interactomics, live-cell imaging, and functional assays in patient-derived neurons will be crucial to fully elucidate the molecular consequences of the Arg87Cys mutation and to identify actionable therapeutic targets. Encouragingly, in silico screening has already identified small molecules with selective affinity for the mutant CYFIP2 protein, supporting the feasibility of pharmacological modulation as a therapeutic approach.^30^ Although still in early stages, these findings lay a promising foundation for the development of targeted interventions in CYFIP2-associated neurodevelopmental disorders.

## Conclusions

Our integrative approach reveals that the Arg87Cys mutation in CYFIP2 drives selective and coordinated alterations in protein interaction networks and protein abundance, with a marked impact on actin cytoskeletal organization. Specifically, comparative cross-linking mass spectrometry identified differential mitochondrial interactions and detected actin–tropomyosin cross-links under the Arg87Cys condition, while label-free proteomics in patient-derived NPCs further demonstrated enrichment of stress fiber-associated proteins. These changes support a model in which the Arg87Cys variant promotes cytoskeletal remodeling and altered cellular architecture, which may underlie key pathogenic mechanisms in CYFIP2-associated neurodevelopmental disorders, underscoring the critical role of cytoskeletal regulation in disease onset and progression. Together, this work provides a mechanistic framework linking CYFIP2 mutation to altered actin dynamics.

## Supplementary Material





## Data Availability

The mass spectrometry proteomics data have been deposited to the ProteomeXchange Consortium via the PRIDE partner repository with the data set identifier PXD 067625.

## Notes

Representative Western blot analysis of transfected cells; chromatograms of the SCX fractionation of cross-linked HEK-293 samples; chromatograms of the LC–MS/MS data acquisition of cross-linked samples; and chromatograms of the LC–MS/MS data acquisition of H1 and SP1C8-derived neural progenitor samples (PDF)
Summary of all the identified CSMs; summary of all the identified peptide pairs; summary of all the identified PPIs; CSMs quantification; and differentially expressed proteins between C8 and H1 cells (XLSX)

## References

[1] Zuberi S. M., Wirrell E., Yozawitz E., Wilmshurst J. M., Specchio N., Riney K., Pressler R., Auvin S., Samia P., Hirsch E., Galicchio S., Triki C., Snead O. C., Wiebe S., Cross J. H., Tinuper P., Scheffer I. E., Perucca E., Moshé S. L., Nabbout R. (2022). ILAE Classification and Definition of Epilepsy Syndromes
with Onset in Neonates and Infants: Position Statement by the ILAE
Task Force on Nosology and Definitions. Epilepsia.

[2] Nakashima M., Kato M., Aoto K., Shiina M., Belal H., Mukaida S., Kumada S., Sato A., Zerem A., Lerman-Sagie T., Lev D., Leong H. Y., Tsurusaki Y., Mizuguchi T., Miyatake S., Miyake N., Ogata K., Saitsu H., Matsumoto N. (2018). De Novo Hotspot Variants in CYFIP2
Cause Early-onset Epileptic Encephalopathy. Ann. Neurol..

[3] Zweier M., Begemann A., McWalter K., Cho M. T., Abela L., Banka S., Behring B., Berger A., Brown C. W., Carneiro M., Chen J., Cooper G. M., Finnila C. R., Guillen Sacoto M. J., Henderson A., Hüffmeier U., Joset P., Kerr B., Lesca G., Leszinski G. S., McDermott J. H., Meltzer M. R., Monaghan K. G., Mostafavi R., Õunap K., Plecko B., Powis Z., Purcarin G., Reimand T., Riedhammer K. M., Schreiber J. M., Sirsi D., Wierenga K. J., Wojcik M. H., Papuc S. M., Steindl K., Sticht H., Rauch A. (2019). Spatially Clustering
de Novo Variants in CYFIP2, Encoding the Cytoplasmic FMRP Interacting
Protein 2, Cause Intellectual Disability and Seizures. Eur. J. Hum. Genet..

[4] Biembengut Í. V., Shigunov P., Frota N. F., Lourenzoni M. R., de Souza T. A. C. B. (2022). Molecular Dynamics of CYFIP2 Protein and Its R87C Variant
Related to Early Infantile Epileptic Encephalopathy. Int. J. Mol. Sci..

[5] Rottner K., Stradal T. E. B., Chen B. (2021). WAVE Regulatory
Complex. Curr. Biol..

[6] Schenck A., Bardoni B., Moro A., Bagni C., Mandel J. L. (2001). A Highly
Conserved Protein Family Interacting with the Fragile X Mental Retardation
Protein (FMRP) and Displaying Selective Interactions with FMRP-Related
Proteins FXR1P and FXR2P. Proc. Natl. Acad.
Sci. U. S. A..

[7] Ding B., Yang S., Schaks M., Liu Y., Brown A. J., Rottner K., Chowdhury S., Chen B. (2022). Structures Reveal a
Key Mechanism of WAVE Regulatory Complex Activation by Rac1 GTPase. Nat. Commun..

[8] Lee Y., Zhang Y., Kang H., Bang G., Kim Y., Kang H. R., Ma R., Jin C., Kim J. Y., Han K. (2020). Epilepsy- and Intellectual Disability-Associated
CYFIP2 Interacts
with Both Actin Regulators and RNA-Binding Proteins in the Neonatal
Mouse Forebrain. Biochem. Biophys. Res. Commun..

[9] Schaks M., Reinke M., Witke W., Rottner K. (2020). Molecular Dissection
of Neurodevelopmental Disorder-Causing Mutations in CYFIP2. Cells.

[10] Markham K., Bai Y., Schmitt-Ulms G. (2007). Co-Immunoprecipitations
Revisited: An Update on Experimental
Concepts and Their Implementation for Sensitive Interactome Investigations
of Endogenous Proteins. Anal. Bioanal. Chem..

[11] O’Reilly F. J., Rappsilber J. (2018). Cross-Linking
Mass Spectrometry: Methods and Applications
in Structural, Molecular and Systems Biology. Nat. Struct. Mol. Biol..

[12] Kurt L. U., Clasen M. A., Biembengut Í.
V., Ruwolt M., Liu F., Gozzo F. C., Lima D. B., Carvalho P. C. (2024). RawVegetable 2.0:
Refining XL-MS Data Acquisition through Enhanced Quality Control. J. Proteome Res..

[13] Santos M. D. M., Lima D. B., Fischer J. S. G., Clasen M. A., Kurt L. U., Camillo-Andrade A. C., Monteiro L. C., de Aquino P. F., Neves-Ferreira A. G. C., Valente R. H., Trugilho M. R. O., Brunoro G. V. F., Souza T. A. C. B., Santos R. M., Batista M., Gozzo F. C., Durán R., Yates J. R., Barbosa V. C., Carvalho P. C. (2022). Simple, Efficient
and Thorough Shotgun Proteomic Analysis
with PatternLab V. Nat. Protoc..

[14] Clasen M. A., Ruwolt M., Wang C., Ruta J., Bogdanow B., Kurt L. U., Zhang Z., Wang S., Gozzo F. C., Chen T., Carvalho P. C., Lima D. B., Liu F. (2024). Proteome-Scale
Recombinant Standards and a Robust High-Speed Search Engine to Advance
Cross-Linking MS-Based Interactomics. Nat. Methods.

[15] Kurt L. U., Clasen M. A., Santos M. D. M., Lyra E. S. B., Santos L. O., Ramos C. H. I., Lima D. B., Gozzo F. C., Carvalho P. C. (2021). Characterizing
Protein Conformers by Cross-Linking Mass Spectrometry and Pattern
Recognition. Bioinformatics.

[16] Tang D., Chen M., Huang X., Zhang G., Zeng L., Zhang G., Wu S., Wang Y. (2023). SRplot: A Free Online
Platform for Data Visualization and Graphing. PLoS One.

[17] Klopfenstein D. V., Zhang L., Pedersen B. S., Ramírez F., Warwick Vesztrocy A., Naldi A., Mungall C. J., Yunes J. M., Botvinnik O., Weigel M., Dampier W., Dessimoz C., Flick P., Tang H. (2018). GOATOOLS: A Python Library for Gene
Ontology Analyses. Sci. Rep..

[18] Szklarczyk D., Kirsch R., Koutrouli M., Nastou K., Mehryary F., Hachilif R., Gable A. L., Fang T., Doncheva N. T., Pyysalo S., Bork P., Jensen L. J., Von Mering C. (2023). The STRING
Database in 2023: Protein-Protein Association Networks and Functional
Enrichment Analyses for Any Sequenced Genome of Interest. Nucleic Acids Res..

[19] Kashina A. S. (2020). Regulation
of Actin Isoforms in Cellular and Developmental Processes. Semin. Cell Dev. Biol..

[20] Schevzov G., Whittaker S. P., Fath T., Lin J. J. C., Gunning P. W. (2011). Tropomyosin
Isoforms and Reagents. Bioarchitecture.

[21] Burridge K., Wittchen E. S. (2013). The Tension Mounts:
Stress Fibers as Force-Generating
Mechanotransducers. J. Cell Biol..

[22] Chen Z., Borek D., Padrick S. B., Gomez T. S., Metlagel Z., Ismail A. M., Umetani J., Billadeau D. D., Otwinowski Z., Rosen M. K. (2010). Structure and Control
of the Actin
Regulatory WAVE Complex. Nature.

[23] Begemann A., Sticht H., Begtrup A., Vitobello A., Faivre L., Banka S., Alhaddad B., Asadollahi R., Becker J., Bierhals T., Brown K. E., Bruel A. L., Brunet T., Carneiro M., Cremer K., Day R., Denommé-Pichon A. S., Dyment D. A., Engels H., Fisher R., Goh E. S., Hajianpour M. J., Haertel L. R. M., Hauer N., Hempel M., Herget T., Johannsen J., Kraus C., Le Guyader G., Lesca G., Mau-Them F. T., McDermott J. H., McWalter K., Meyer P., Õunap K., Popp B., Reimand T., Riedhammer K. M., Russo M., Sadleir L. G., Saenz M., Schiff M., Schuler E., Syrbe S., Van der Ven A. T., Verloes A., Willems M., Zweier C., Steindl K., Zweier M., Rauch A. (2021). New Insights into the Clinical and
Molecular Spectrum of the Novel CYFIP2-Related Neurodevelopmental
Disorder and Impairment of the WRC-Mediated Actin Dynamics. Genet. Med..

[24] Beck M. R., Dixon R. D. S., Goicoechea S. M., Murphy G. S., Brungardt J. G., Beam M. T., Srinath P., Patel J., Mohiuddin J., Otey C. A., Campbell S. L. (2013). Structure
and Function of Palladin’s
Actin Binding Domain. J. Mol. Biol..

[25] Lin J. J., Li Y., Eppinga R. D., Wang Q., Jin J. P. (2009). Chapter 1: Roles
of Caldesmon in Cell Motility and Actin Cytoskeleton Remodeling. Int. Rev. Cell Mol. Biol..

[26] Petit M. M. R., Meulemans S. M. P., Van de Ven W. J. M. (2003). The
Focal Adhesion and Nuclear Targeting Capacity of the LIM-Containing
Lipoma-Preferred Partner (LPP) Protein. J. Biol.
Chem..

[27] Yi J., Beckerle M. C. (1998). The HumanTRIP6Gene
Encodes a LIM Domain Protein and
Maps to Chromosome 7q22, a Region Associated with Tumorigenesis. Genomics.

[28] Cingolani L. A., Goda Y. (2008). Actin in Action: The Interplay between the Actin Cytoskeleton and
Synaptic Efficacy. Nat. Rev. Neurosci..

[29] Zhang Y., Kang H. R., Jun Y., Kang H., Bang G., Ma R., Ju S., Yoon D. E., Kim Y., Kim K., Kim J. Y., Han K. (2024). Neurodevelopmental Disorder-Associated
CYFIP2 Regulates Membraneless Organelles and EIF2α Phosphorylation
via Protein Interactors and Actin Cytoskeleton. Hum. Mol. Genet..

[30] Venturi
Biembengut Í., de Castro Andreassa E., de Souza T. A. C. B. (2024). Identification
of CYFIP2 Arg87Cys Ligands via In Silico and In Vitro Approaches. Biomedicines.

