# Supplementary material for: The CYFIP2 Arg87Cys Mutation Reshapes the Interactome and Alters Stress Fiber-Associated Proteins
Source: ACS Omega. 2026 Oct 12;11(42):64371–8. doi: 10.1021/acsomega.5c12192 (PMC13718196; doi:10.1021/acsomega.5c12192)
Supplement: Supplementary file 1 [file ao5c12192_si_001.pdf]

Supporting Information

The CYFIP2 Arg87Cys Mutation Reshapes the Interactome and Alters Stress Fiber-Associated Proteins

Ísis V. Biembengut[1], Emanuella de C. Andreassa[1], Marlon D. M. dos Santos[1], Isabelle L. Z.Silva[2], Louise U. Kurt[1], Patrícia Shigunov[2], Paulo C. Carvalho[1], Tatiana A. C. B. de Souza[1].*

1. *Laboratory for Structural and Computational Proteomics, Instituto Carlos Chagas, Fundação Oswaldo Cruz (Fiocruz), Curitiba, PR 81350-010, Brazil*

2. *Stem Cell Basic Biology Laboratory, Instituto Carlos Chagas, Fundação Oswaldo Cruz (Fiocruz), Curitiba, PR 81350-010, Brazil*

*Corresponding author: Tatiana A. C. B de Souza, Tatiana.brasil@fiocruz.br

Contents

Figure S1. Representative Western blot analysis of transfected cells

Figure S2. Chromatograms of the SCX fractionation of cross-linked HEK-293 samples

Figure S3. Chromatograms of the LC–MS/MS data acquisition of cross-linked samples

Figure S4. Chromatograms of the LC–MS/MS data acquisition of H1 and SP1C8-derived neural progenitor samples

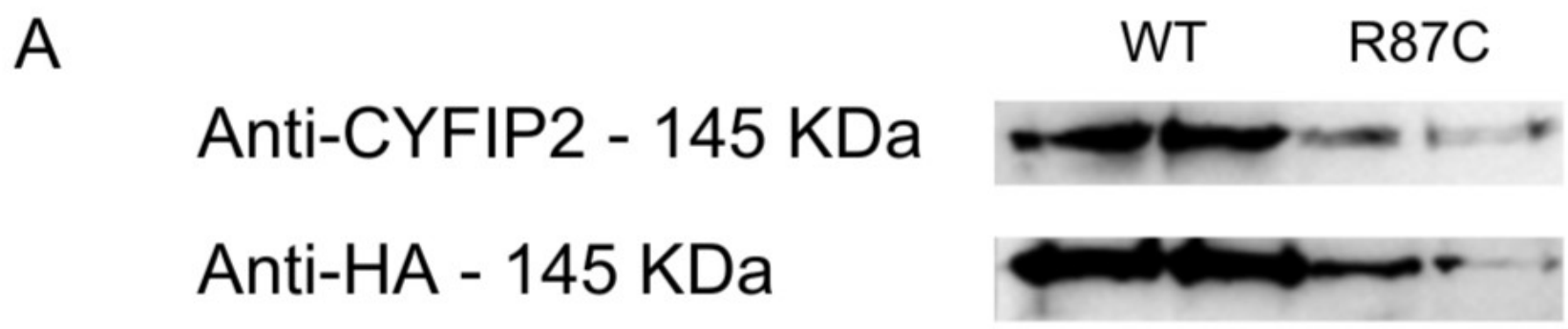

Figure S1. Representative Western blot analysis of transfected cells. (A) HEK-293 cells transfected with CYFIP2 WT or Arg87Cys (R87C) plasmids; immunoblots probed with anti-CYFIP2 (145 kDa) and anti-HA (145 kDa) antibodies.

A

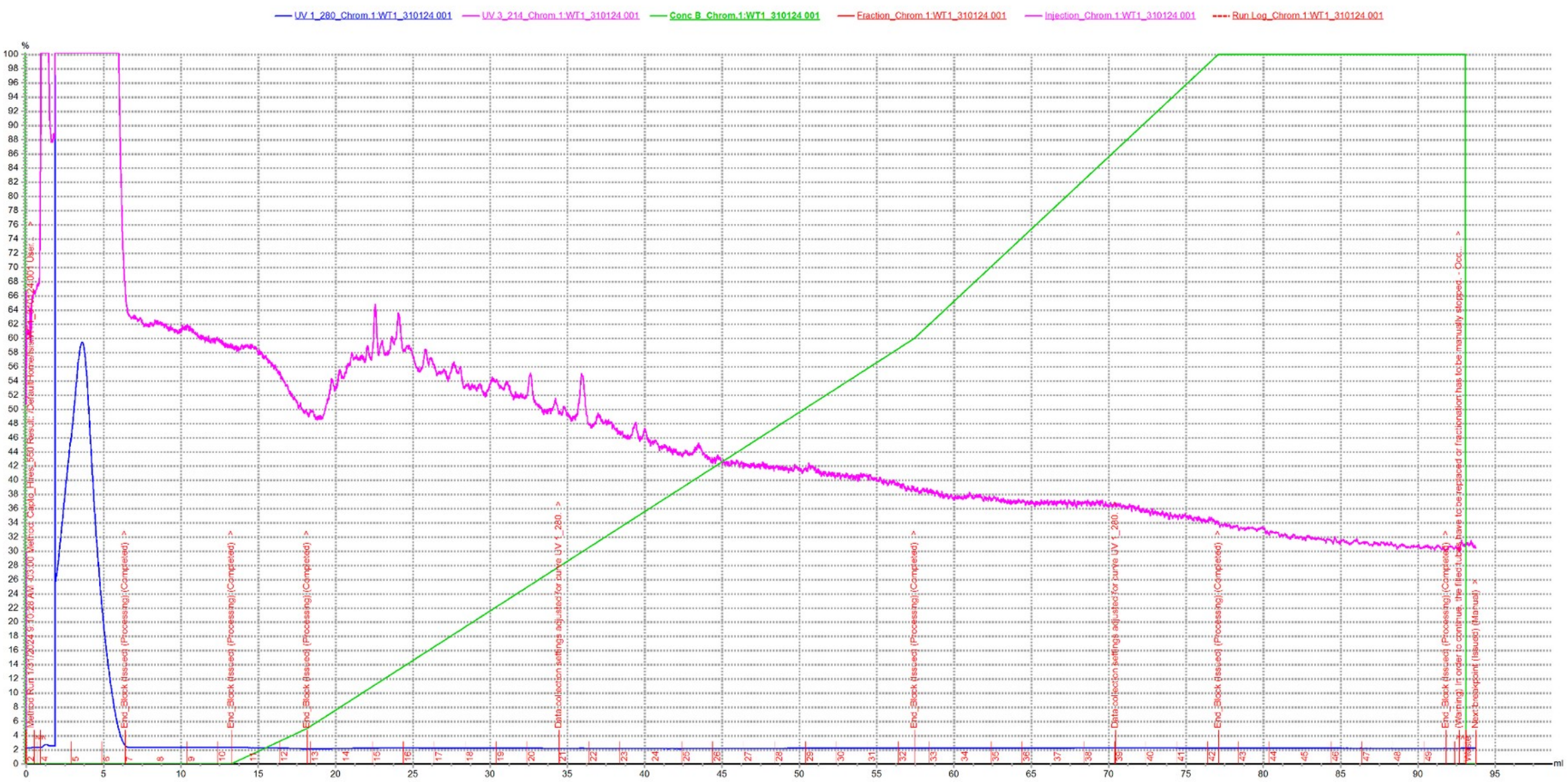

UV 1_280_Chrom.1:WT1_310124 001
UV 3_214_Chrom.1:WT1_310124 001
Conc B_Chrom.1:WT1_310124 001
Fraction_Chrom.1:WT1_310124 001
Injection_Chrom.1:WT1_310124 001
Run Log_Chrom.1:WT1_310124 001
%
ml

B

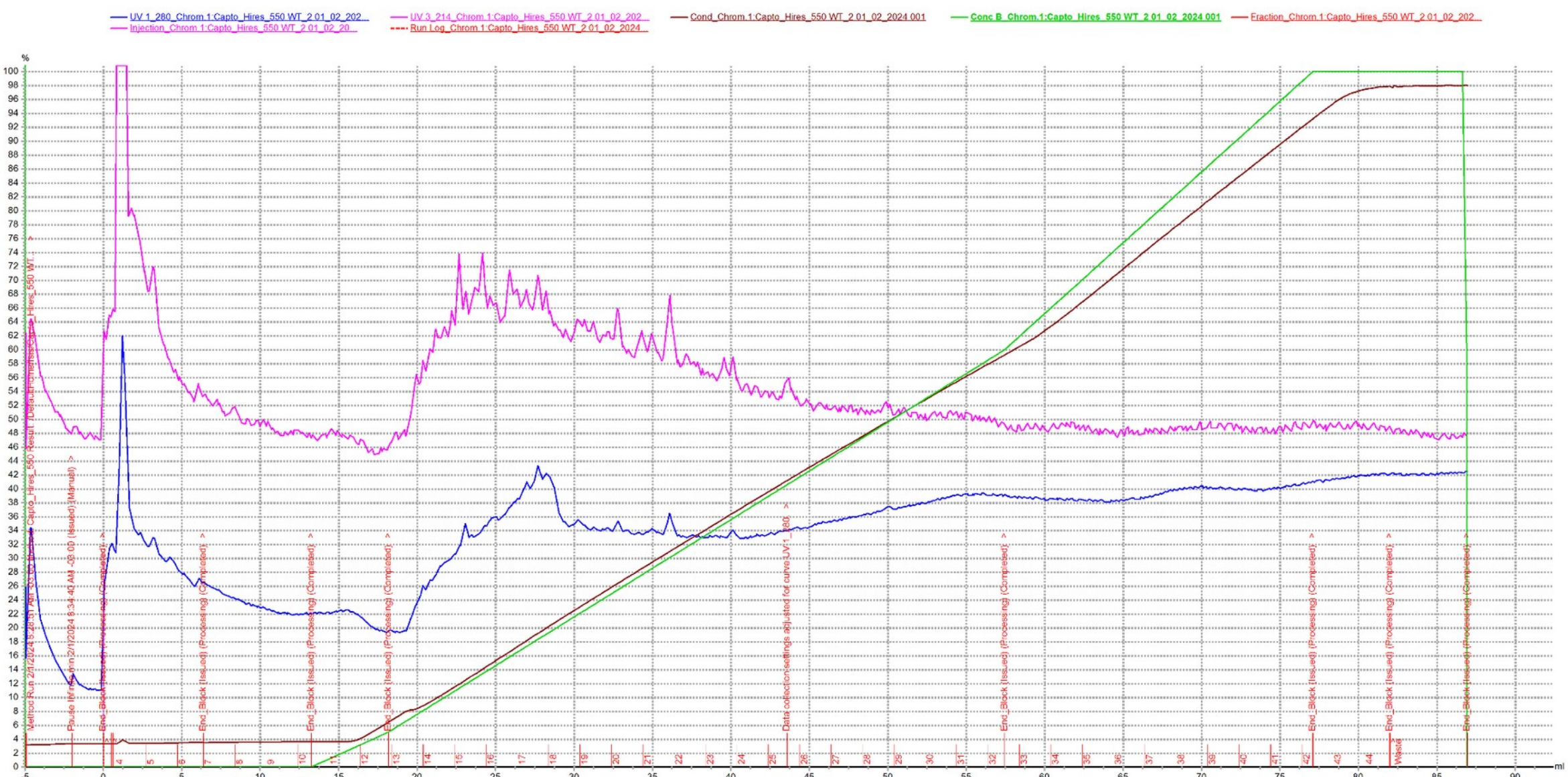

UV 1_280_Chrom.1:Capto_Hires_550 WT_2 01_02_202...
UV 3_214_Chrom.1:Capto_Hires_550 WT_2 01_02_202...
Cond_Chrom.1:Capto_Hires_550 WT_2 01_02_2024 001
Conc B_Chrom.1:Capto_Hires_550 WT_2 01_02_2024 001
Fraction_Chrom.1:Capto_Hires_550 WT_2 01_02_202...
Injection_Chrom.1:Capto_Hires_550 WT_2 01_02_20...
Run Log_Chrom.1:Capto_Hires_550 WT_2 01_02_2024...
%
ml

C

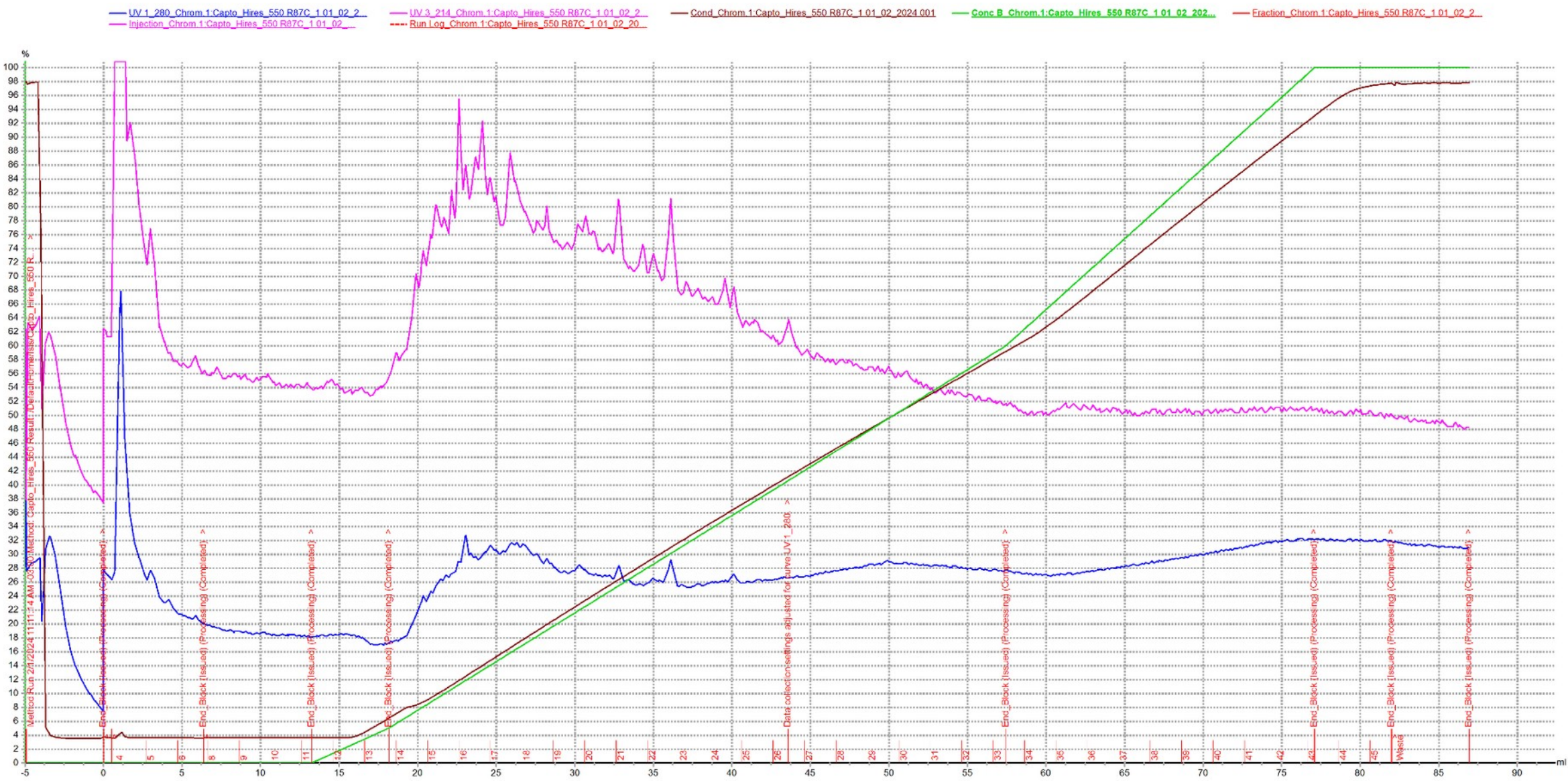
UV 1_280_Chrom.1:Capto_Hires_550 R87C_1.01_02_2...
Injection_Chrom.1:Capto_Hires_550 R87C_1.01_02_...
UV 3_214_Chrom.1:Capto_Hires_550 R87C_1.01_02_2...
Run Log_Chrom.1:Capto_Hires_550 R87C_1.01_02_20...
Cond_Chrom.1:Capto_Hires_550 R87C_1.01_02_2024.001
Conc B_Chrom.1:Capto_Hires_550 R87C_1.01_02_202...
Fraction_Chrom.1:Capto_Hires_550 R87C_1.01_02_2...
%
ml

D

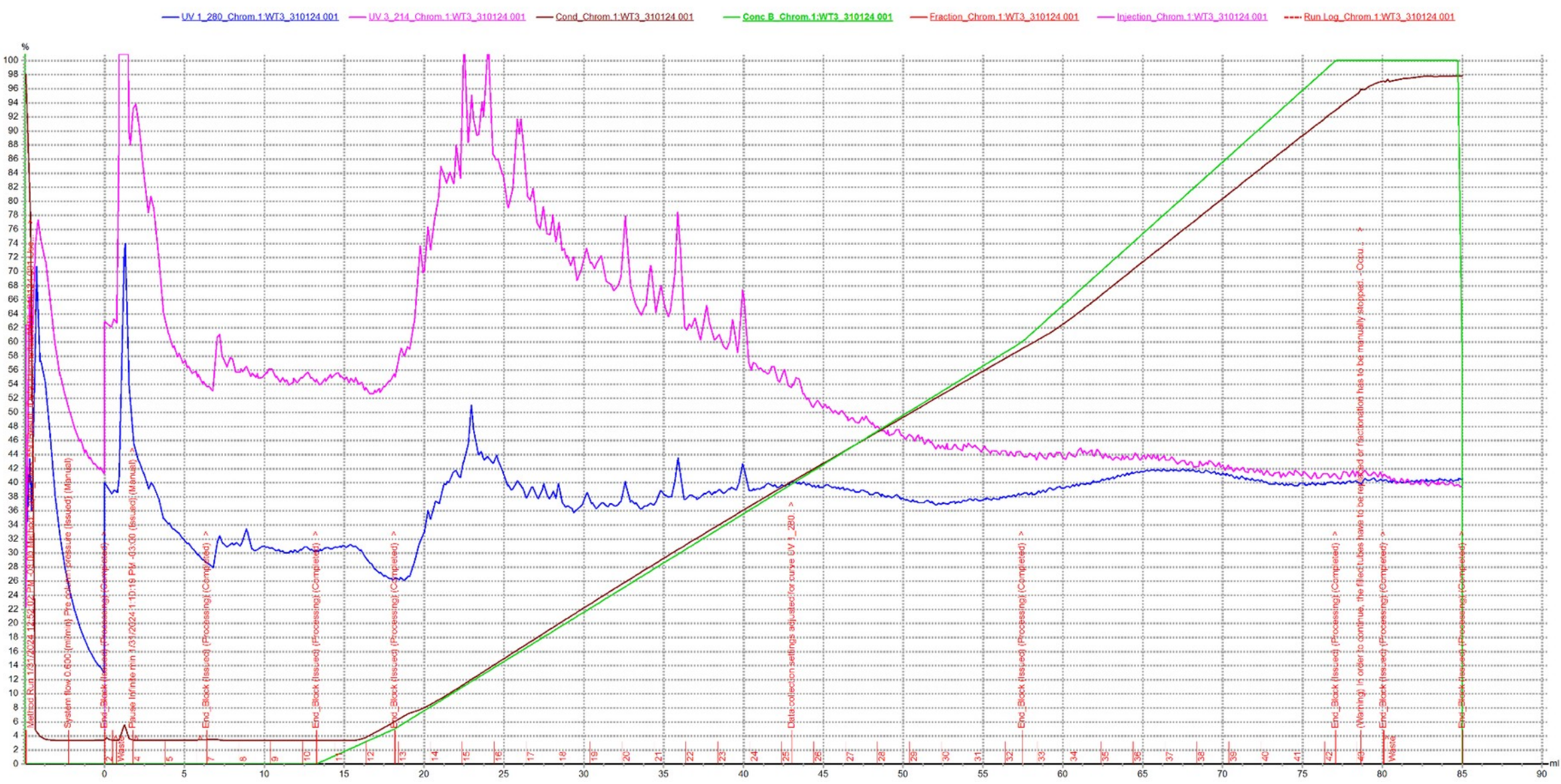
UV 1_280_Chrom.1:WT3_310124.001
UV 3_214_Chrom.1:WT3_310124.001
Cond_Chrom.1:WT3_310124.001
Conc B_Chrom.1:WT3_310124.001
Fraction_Chrom.1:WT3_310124.001
Injection_Chrom.1:WT3_310124.001
Run Log_Chrom.1:WT3_310124.001
%
ml

E

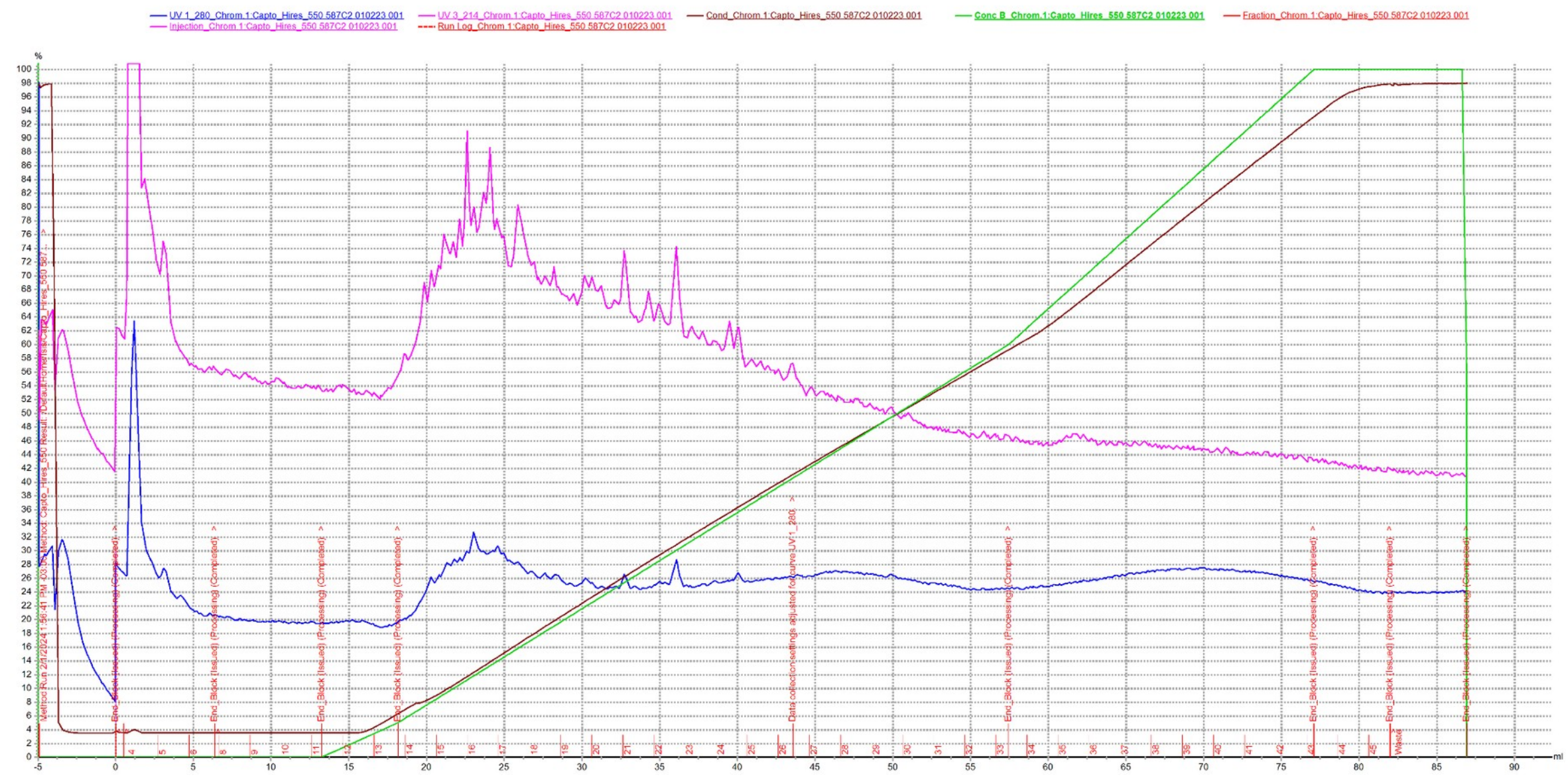

F

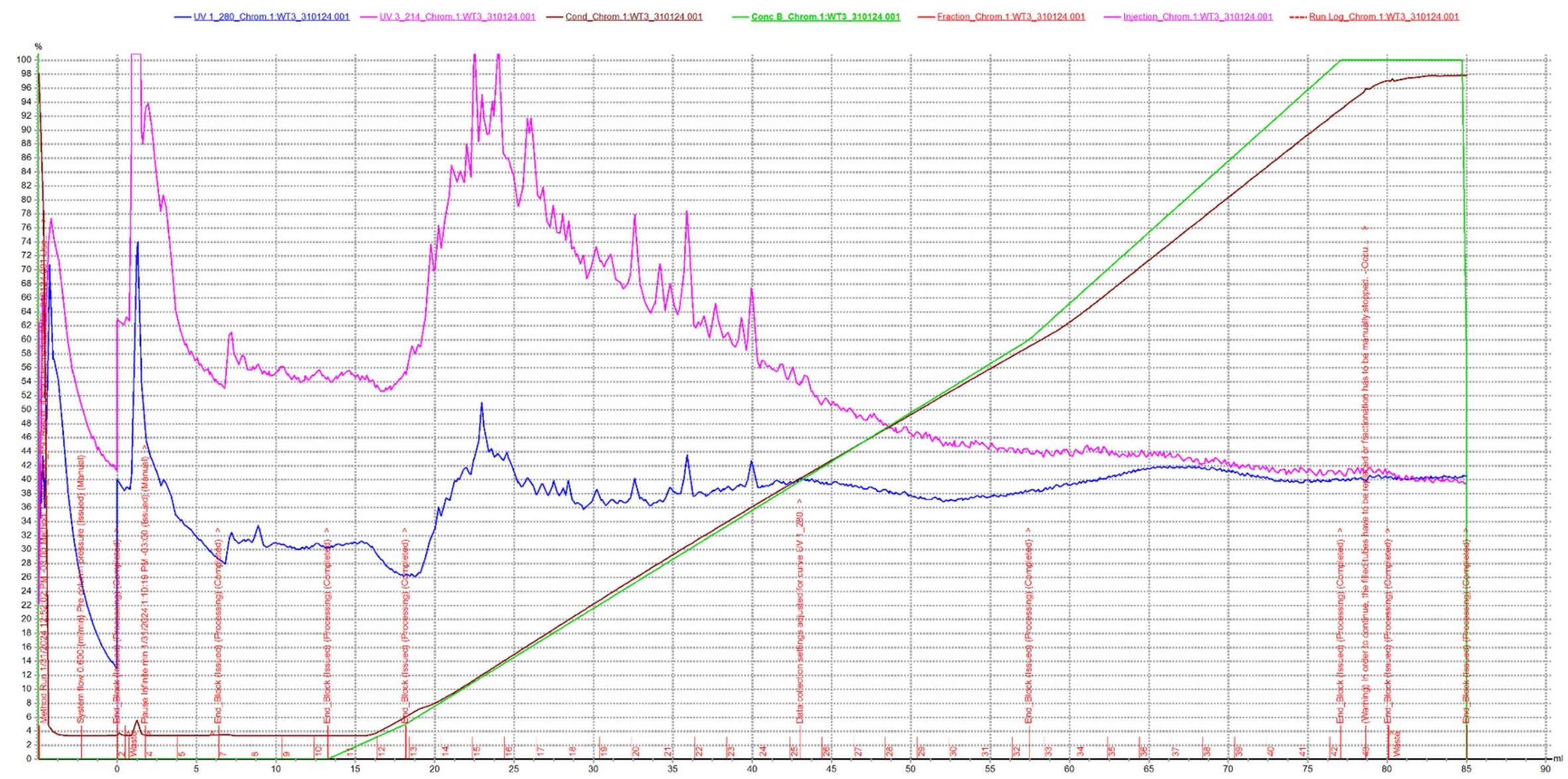

Figure S2. Chromatograms of the SCX fractionation of cross-linked HEK-293 samples (data from ÄKTA UPC 100 system). (A) HEK-293 transfected with wild-type CYFIP2 plasmid, replica 1; (B) HEK-293 transfected with wild-type CYFIP2 plasmid, replica 2; (C) HEK-293 transfected with wild-type CYFIP2 plasmid, replica 3; (D) HEK-293 transfected with Arg87Cys mutant CYFIP2 plasmid, replica 1; (E) HEK-293 transfected with Arg87Cys mutant CYFIP2 plasmid, replica 2; (F) HEK-293 transfected with Arg87Cys mutant CYFIP2 plasmid, replica 3.

A

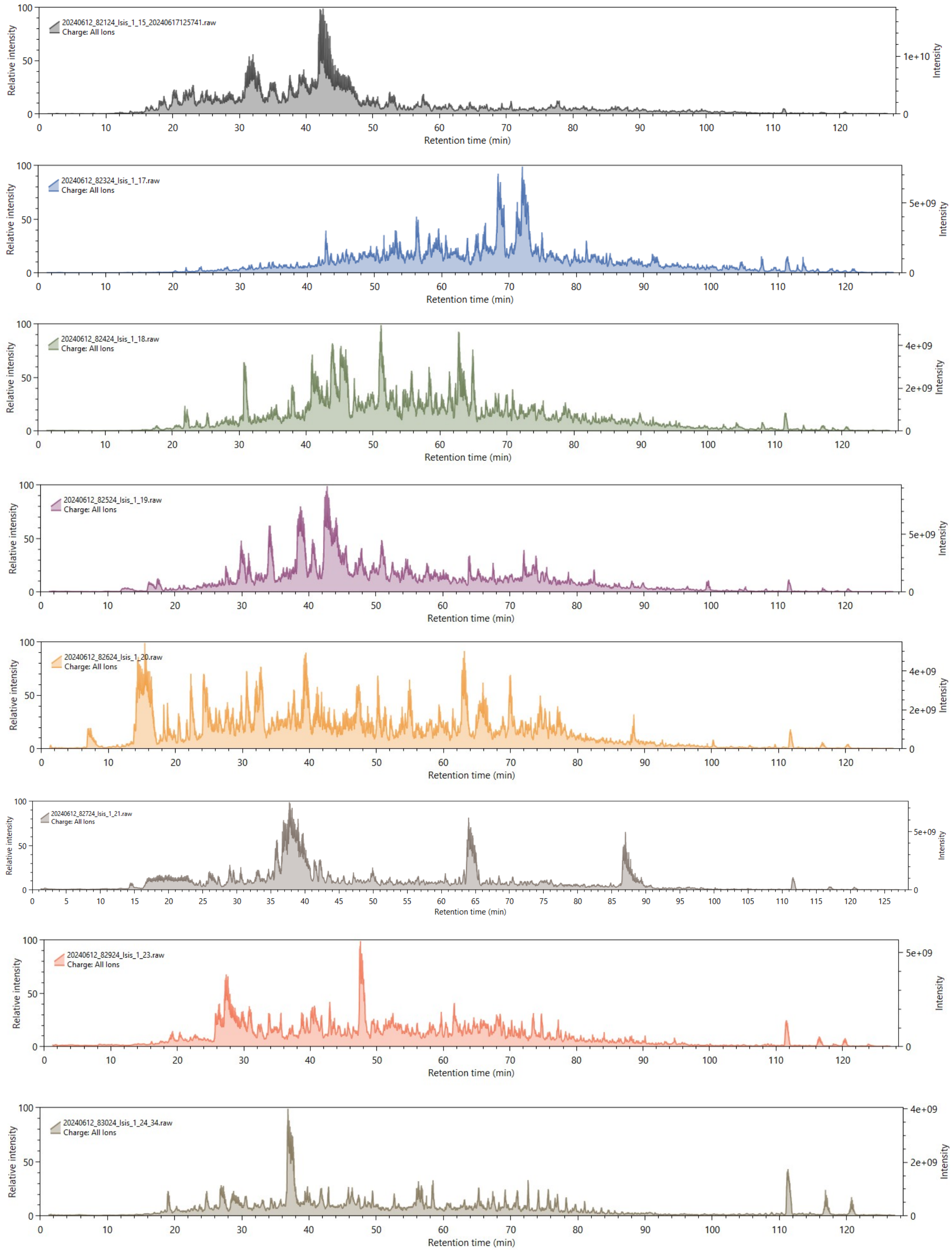
20240612_82124_Isis_1_15_20240617125741.raw
Charge: All Ions
20240612_82324_Isis_1_17.raw
Charge: All Ions
20240612_82424_Isis_1_18.raw
Charge: All Ions
20240612_82524_Isis_1_19.raw
Charge: All Ions
20240612_82624_Isis_1_20.raw
Charge: All Ions
20240612_82724_Isis_1_21.raw
Charge: All Ions
20240612_82924_Isis_1_23.raw
Charge: All Ions
20240612_83024_Isis_1_24_34.raw
Charge: All Ions
Relative intensity
Intensity
Retention time (min)

B

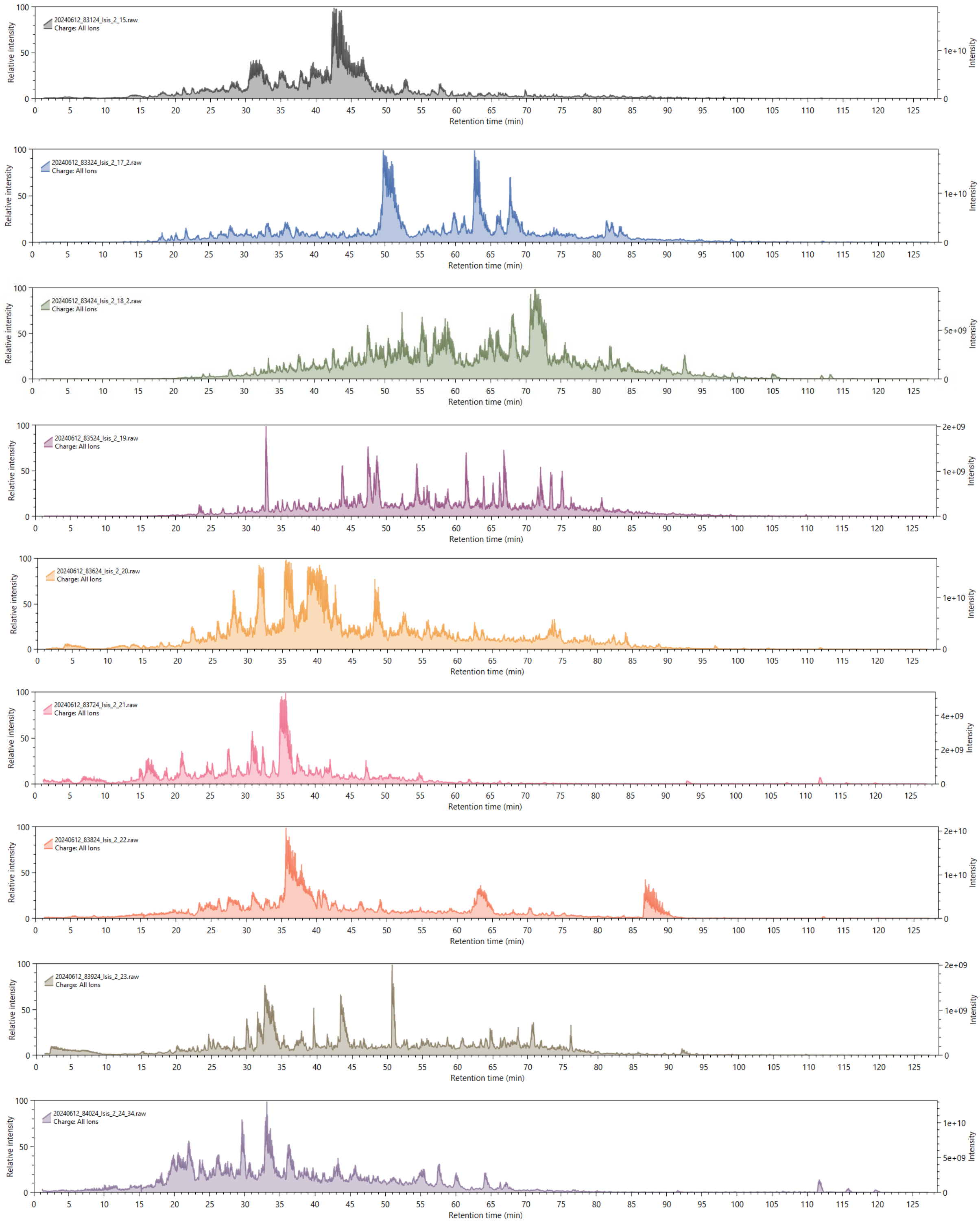
20240612_83124_Isis_2_15.raw
Charge: All Ions
20240612_83324_Isis_2_17_2.raw
Charge: All Ions
20240612_83424_Isis_2_18_2.raw
Charge: All Ions
20240612_83524_Isis_2_19.raw
Charge: All Ions
20240612_83624_Isis_2_20.raw
Charge: All Ions
20240612_83724_Isis_2_21.raw
Charge: All Ions
20240612_83824_Isis_2_22.raw
Charge: All Ions
20240612_83924_Isis_2_23.raw
Charge: All Ions
20240612_84024_Isis_2_24_34.raw
Charge: All Ions
Relative intensity
Retention time (min)
Intensity

C

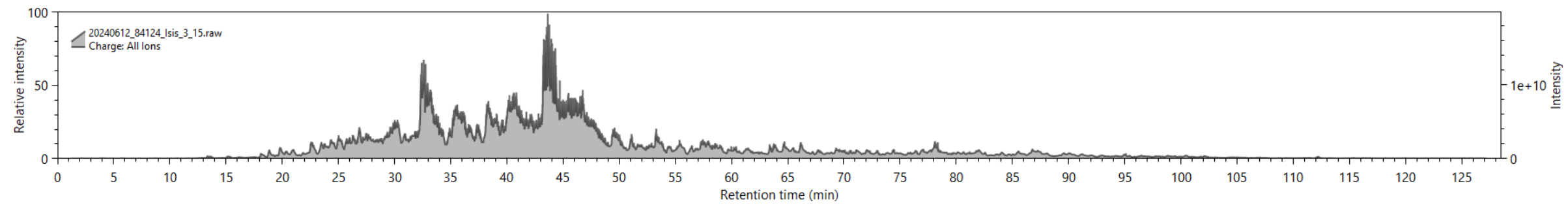
20240612_84124_Isis_3_15.raw
Charge: All Ions
Relative intensity
Retention time (min)
Intensity

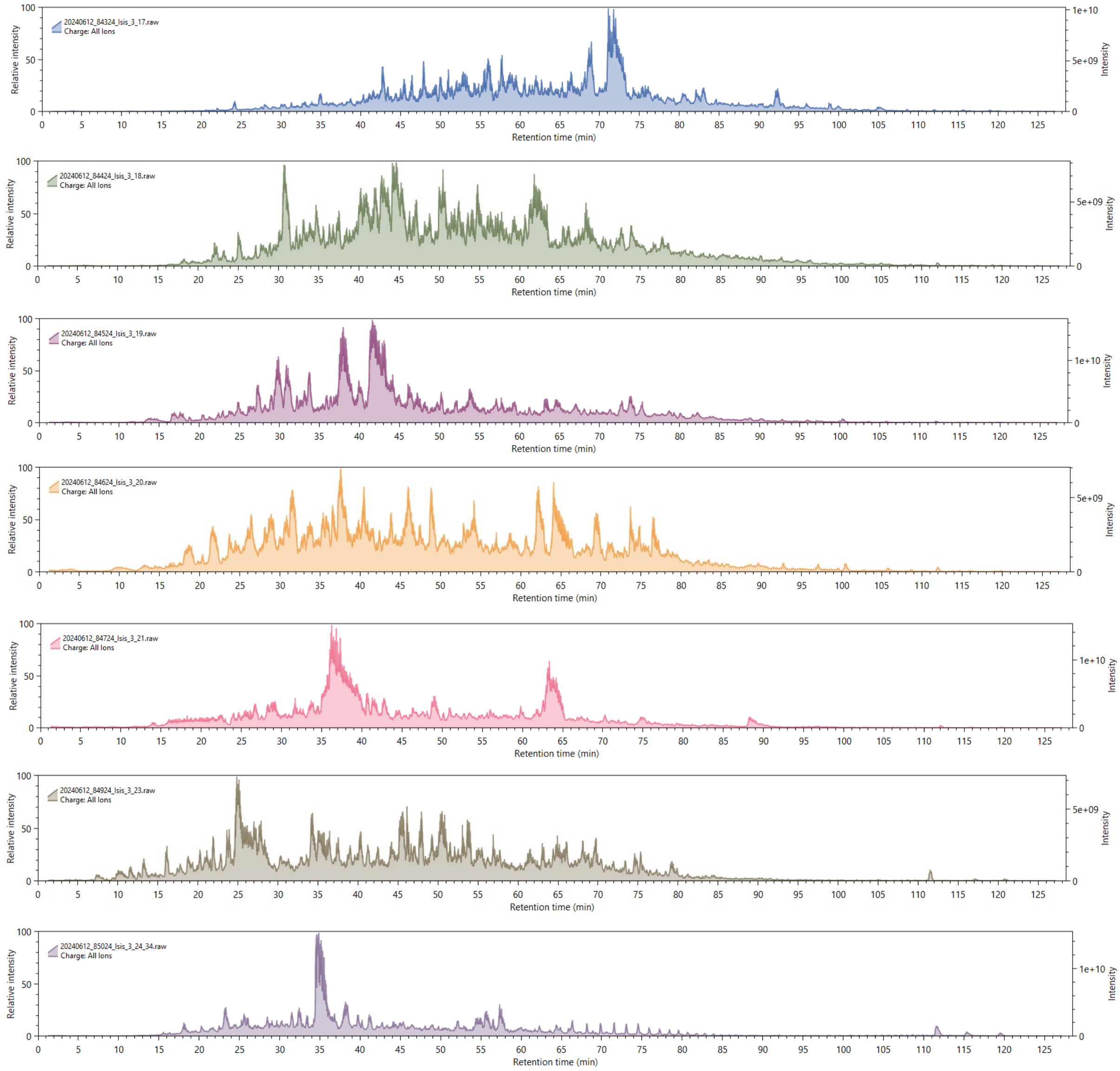
20240612_84324_Isis_3_17.raw
Charge: All Ions
20240612_84424_Isis_3_18.raw
Charge: All Ions
20240612_84524_Isis_3_19.raw
Charge: All Ions
20240612_84624_Isis_3_20.raw
Charge: All Ions
20240612_84724_Isis_3_21.raw
Charge: All Ions
20240612_84924_Isis_3_23.raw
Charge: All Ions
20240612_85024_Isis_3_24_34.raw
Charge: All Ions
Relative intensity
Intensity
Retention time (min)

D

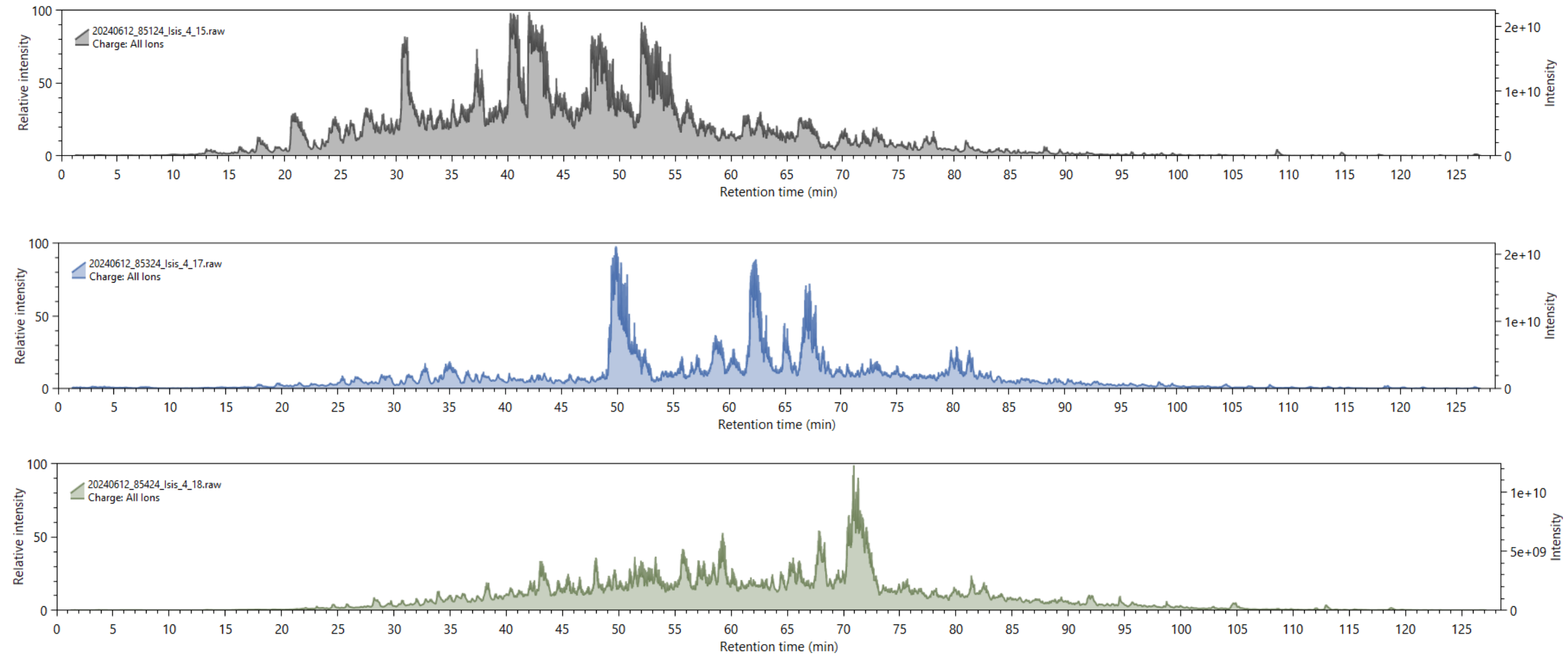
20240612_85124_Isis_4_15.raw
Charge: All Ions
20240612_85324_Isis_4_17.raw
Charge: All Ions
20240612_85424_Isis_4_18.raw
Charge: All Ions
Relative intensity
Intensity
Retention time (min)

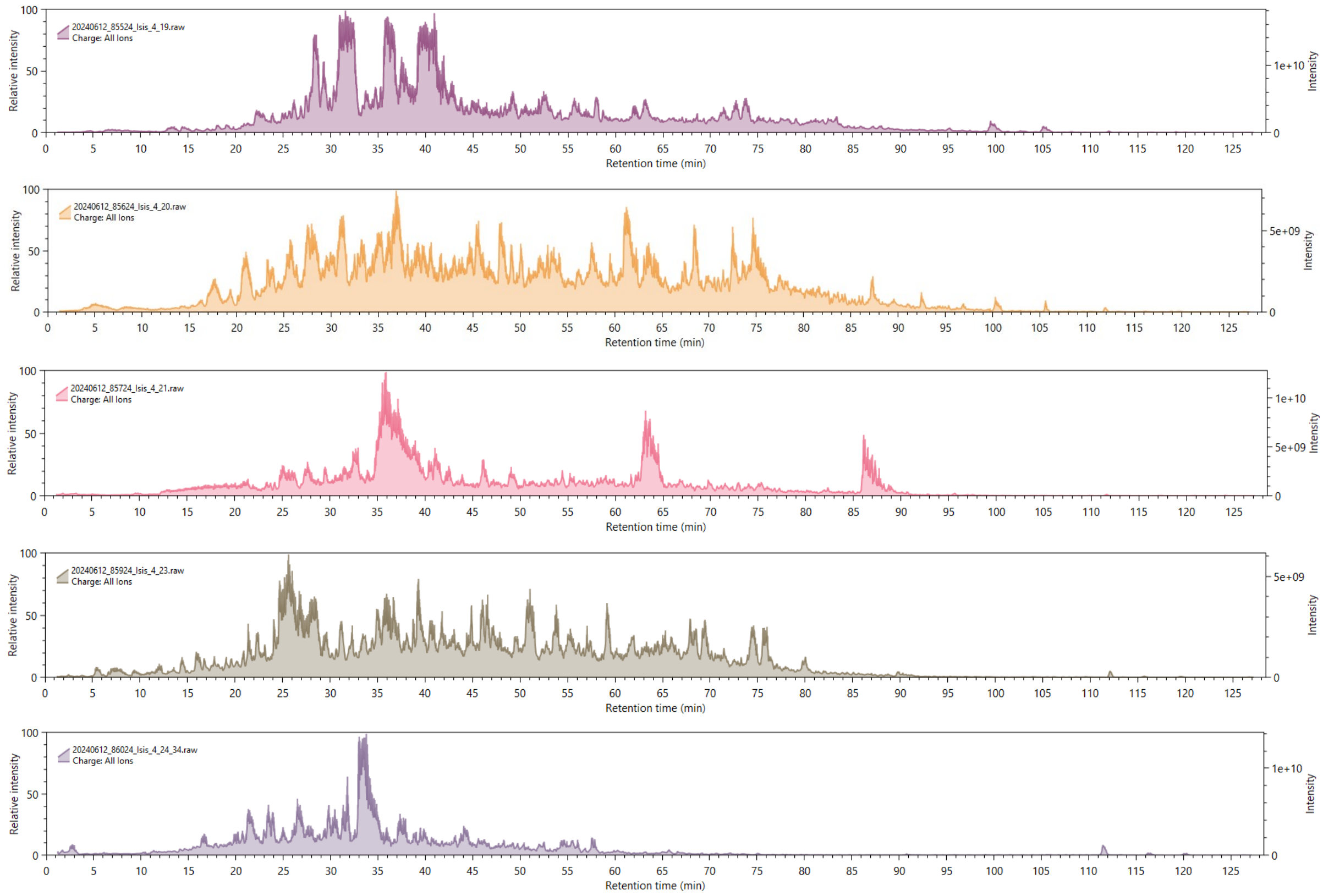
20240612_85524_Isis_4_19.raw
Charge: All Ions
20240612_85624_Isis_4_20.raw
Charge: All Ions
20240612_85724_Isis_4_21.raw
Charge: All Ions
20240612_85924_Isis_4_23.raw
Charge: All Ions
20240612_86024_Isis_4_24_34.raw
Charge: All Ions
Relative intensity
Intensity
Retention time (min)

E

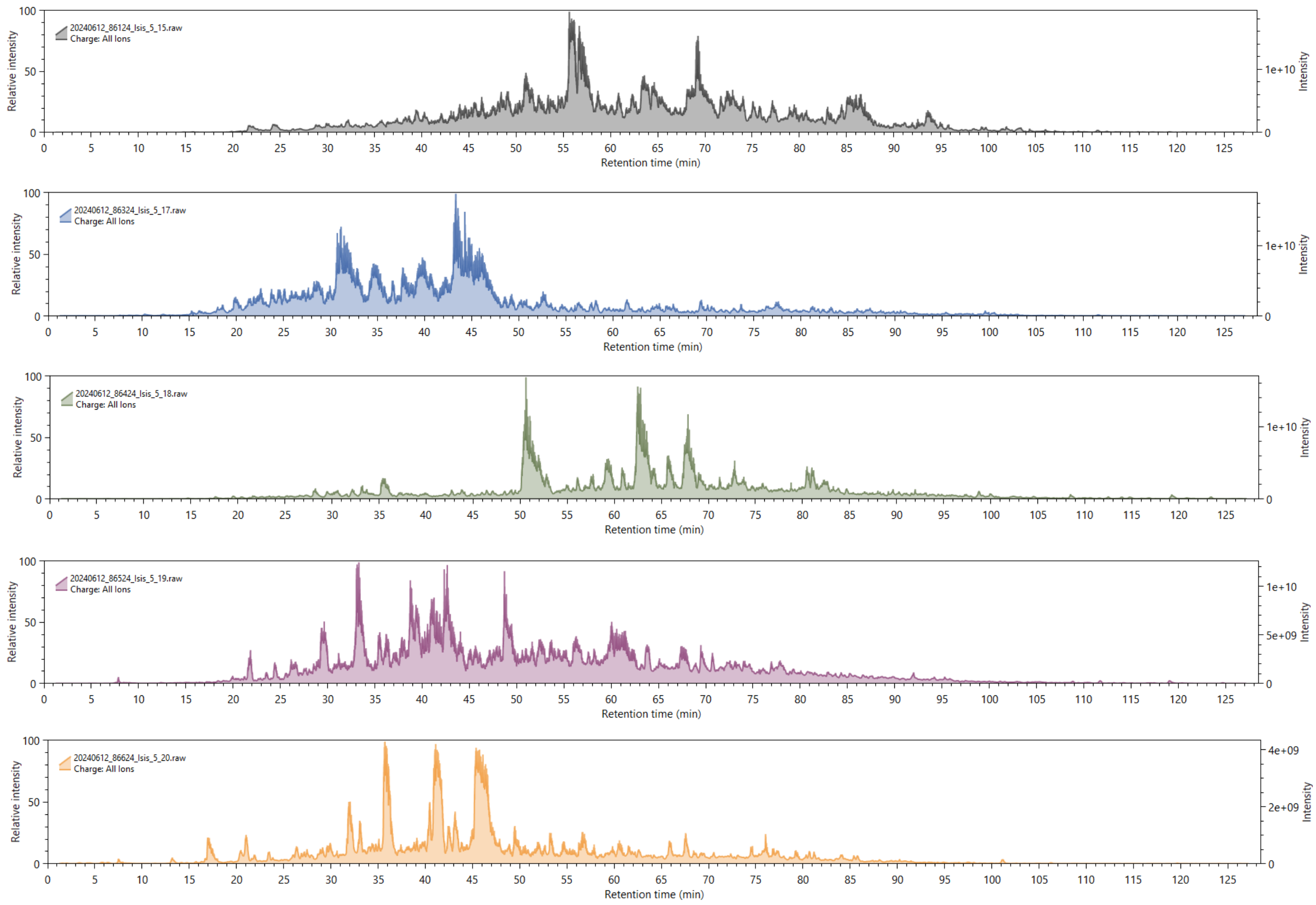
20240612_86124_Isis_5_15.raw
Charge: All Ions
20240612_86324_Isis_5_17.raw
Charge: All Ions
20240612_86424_Isis_5_18.raw
Charge: All Ions
20240612_86524_Isis_5_19.raw
Charge: All Ions
20240612_86624_Isis_5_20.raw
Charge: All Ions
Relative intensity
Intensity
Retention time (min)

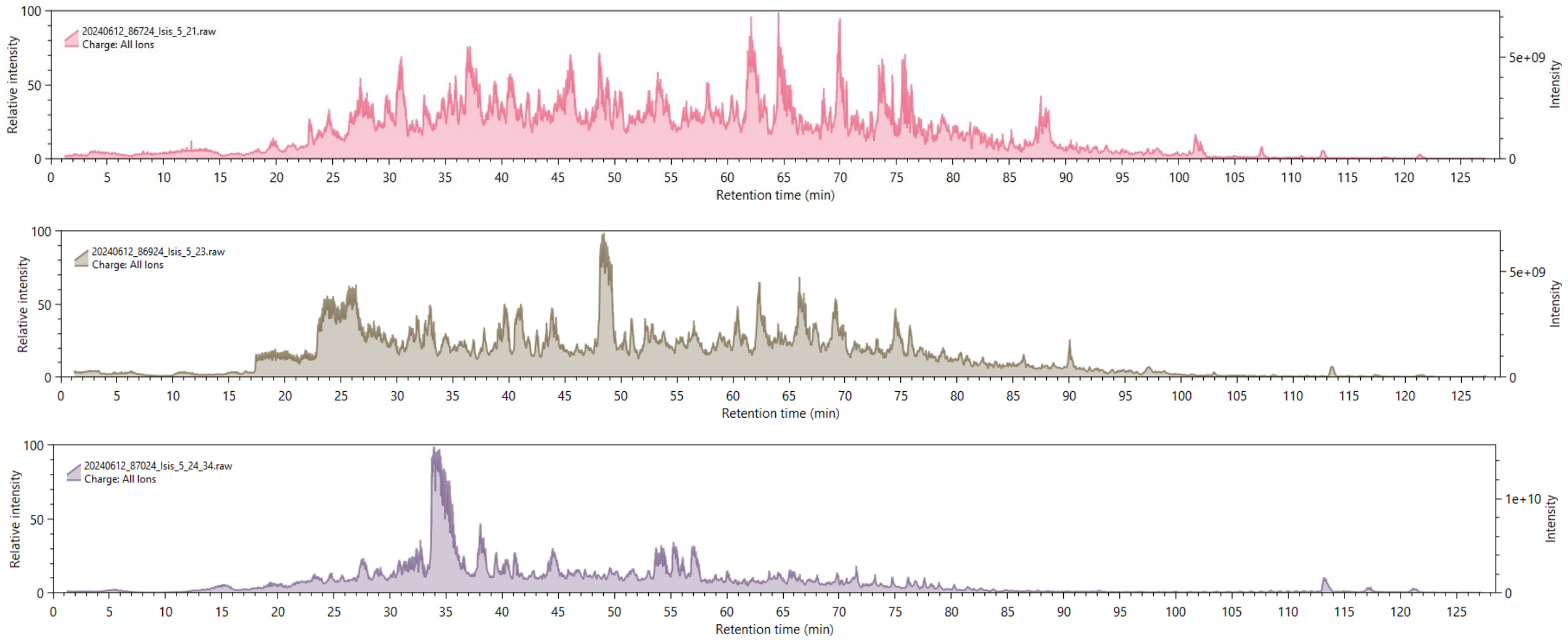
20240612_86724_Isis_5_21.raw
Charge: All Ions
20240612_86924_Isis_5_23.raw
Charge: All Ions
20240612_87024_Isis_5_24_34.raw
Charge: All Ions
Relative intensity
Intensity
Retention time (min)

F

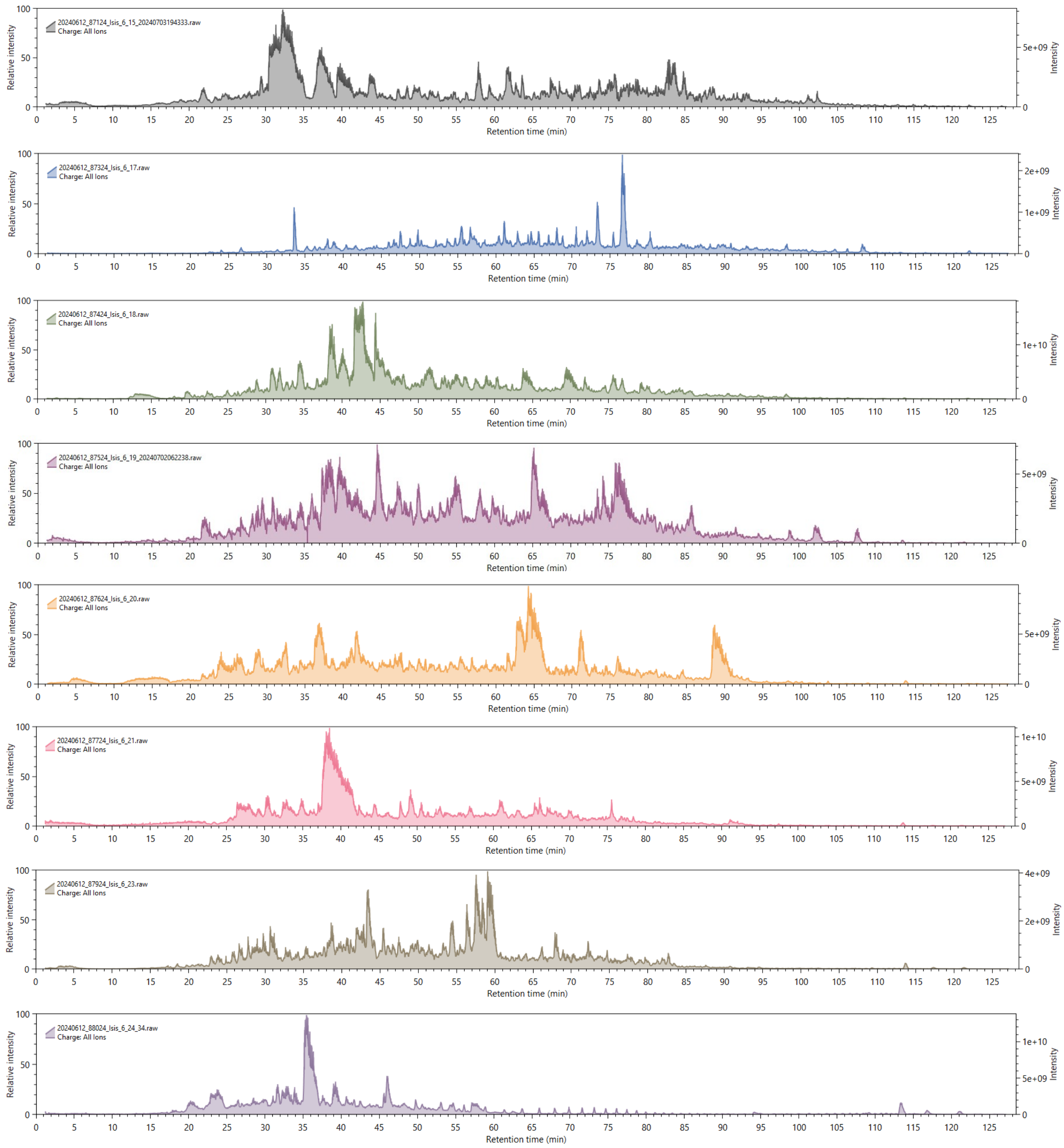
20240612_87124_Isis_6_15_20240703194333.raw
Charge: All Ions
20240612_87324_Isis_6_17.raw
Charge: All Ions
20240612_87424_Isis_6_18.raw
Charge: All Ions
20240612_87524_Isis_6_19_20240702062238.raw
Charge: All Ions
20240612_87624_Isis_6_20.raw
Charge: All Ions
20240612_87724_Isis_6_21.raw
Charge: All Ions
20240612_87924_Isis_6_23.raw
Charge: All Ions
20240612_88024_Isis_6_24_34.raw
Charge: All Ions
Relative intensity
Intensity
Retention time (min)

Figure S3. Chromatograms of the LC–MS/MS data acquisition of cross-linked samples. (A) HEK-293 transfected with wild-type CYFIP2 plasmid, replica 1; (B) HEK-293 transfected with wild-type CYFIP2 plasmid, replica 2; (C) HEK-293 transfected with wild-type CYFIP2 plasmid, replica 3; (D) HEK-293 transfected with Arg87Cys mutant CYFIP2 plasmid, replica 1; (E) HEK-293 transfected with Arg87Cys mutant CYFIP2 plasmid, replica 2; (F) HEK-293 transfected with Arg87Cys mutant CYFIP2 plasmid, replica 3.

A

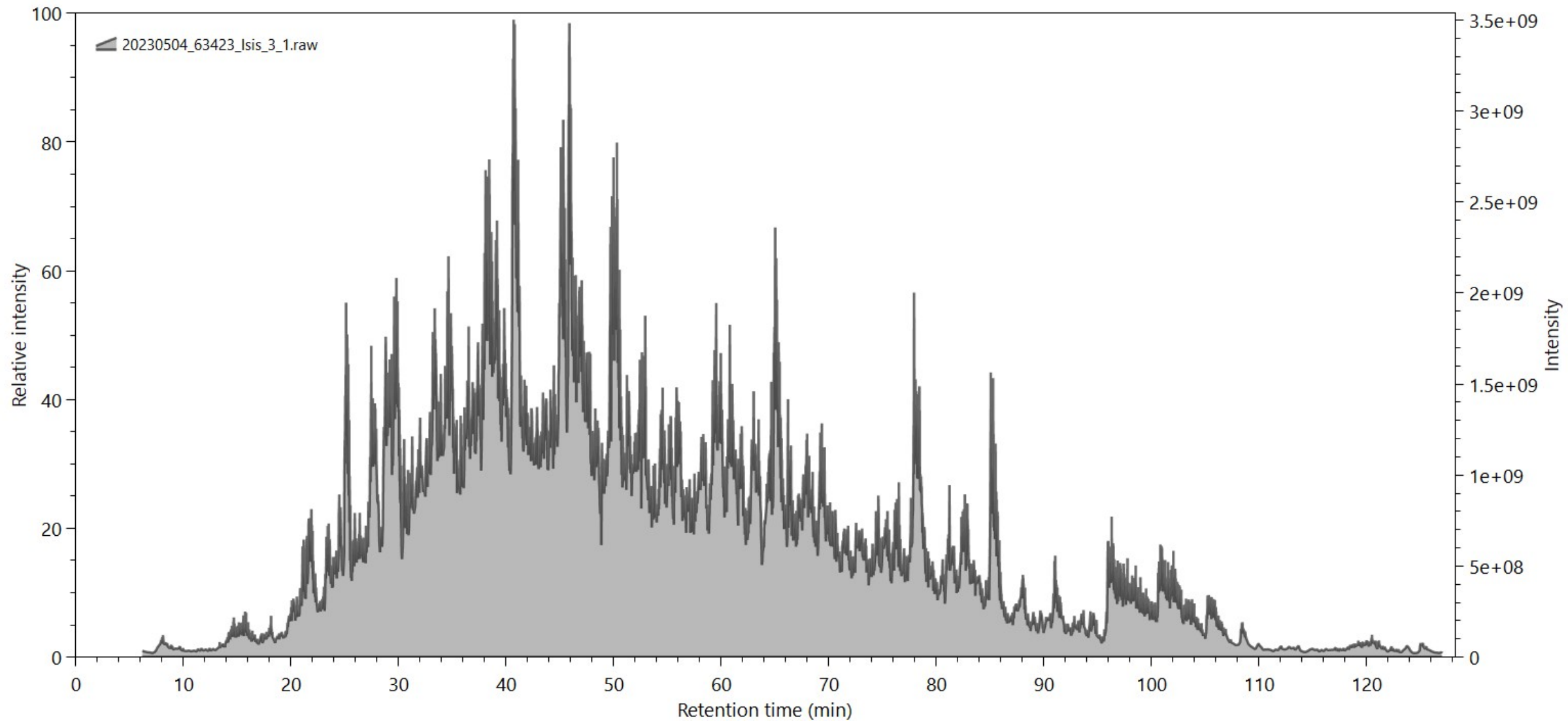
20230504_63423_Isis_3_1.raw
Relative intensity
Intensity
Retention time (min)
0
10
20
30
40
50
60
70
80
90
100
110
120
20
40
60
80
100
5e+08
1e+09
1.5e+09
2e+09
2.5e+09
3e+09
3.5e+09

B

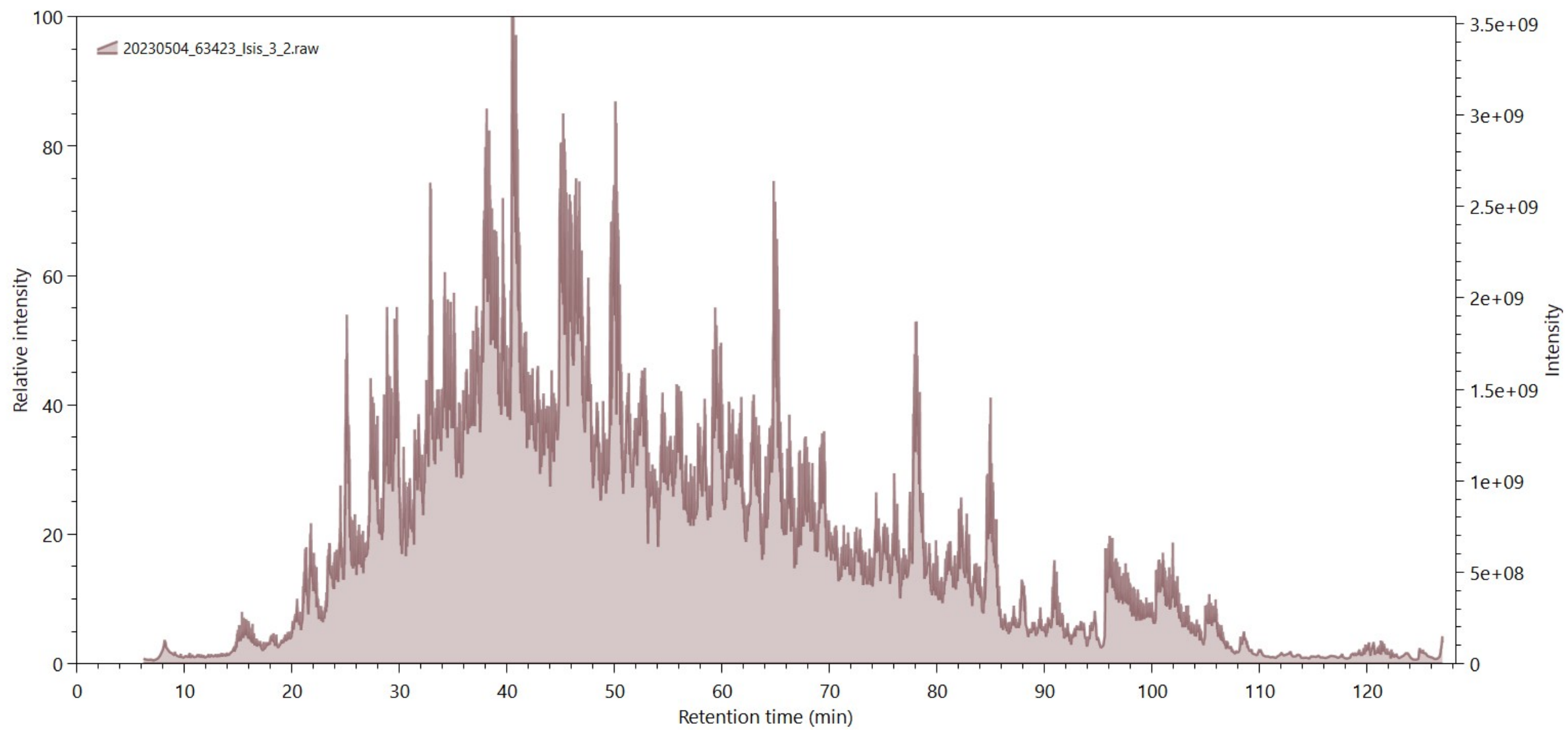
20230504_63423_Isis_3_2.raw
Relative intensity
Intensity
Retention time (min)
0
10
20
30
40
50
60
70
80
90
100
110
120
20
40
60
80
100
5e+08
1e+09
1.5e+09
2e+09
2.5e+09
3e+09
3.5e+09

C

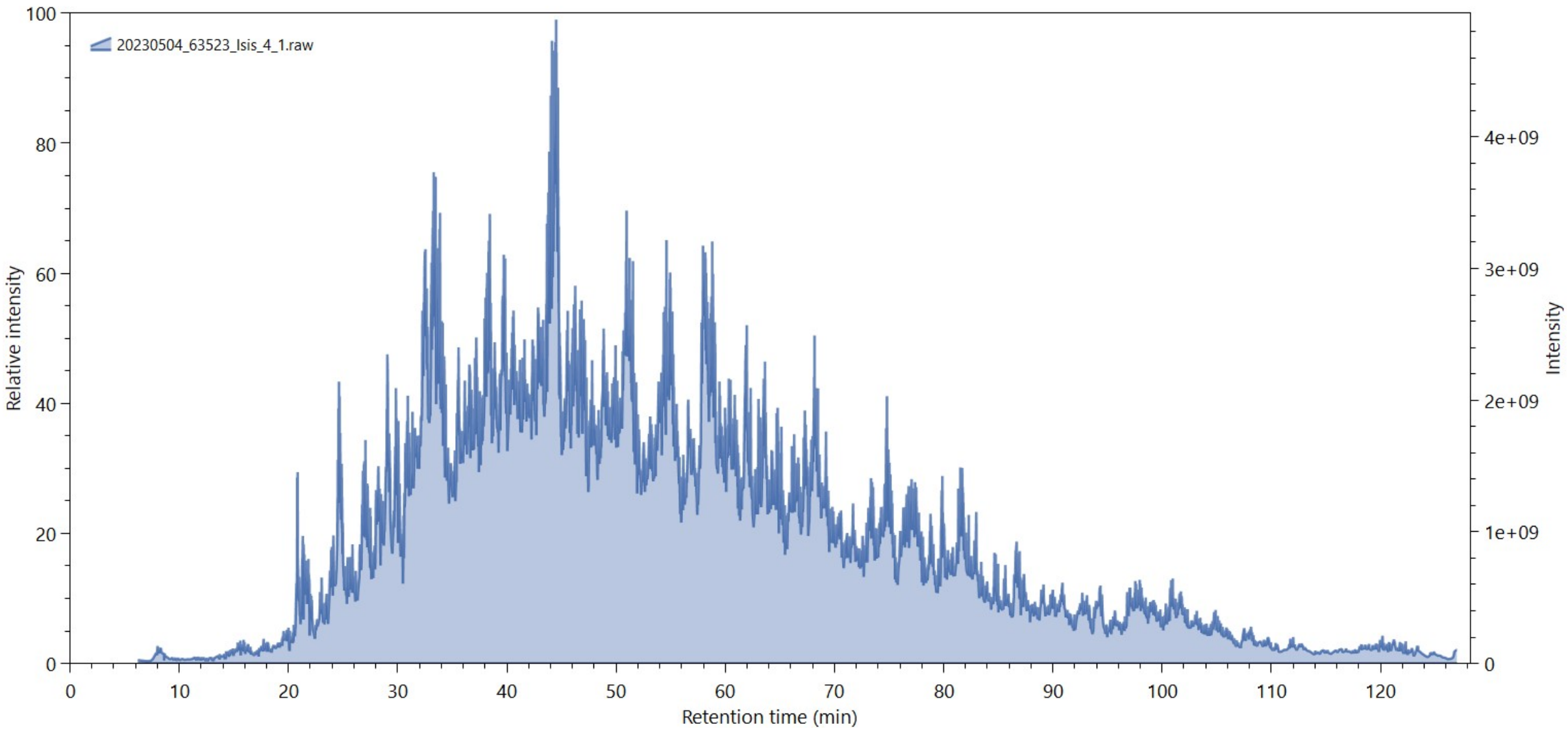
20230504_63523_Isis_4_1.raw
Relative intensity
Intensity
Retention time (min)

D

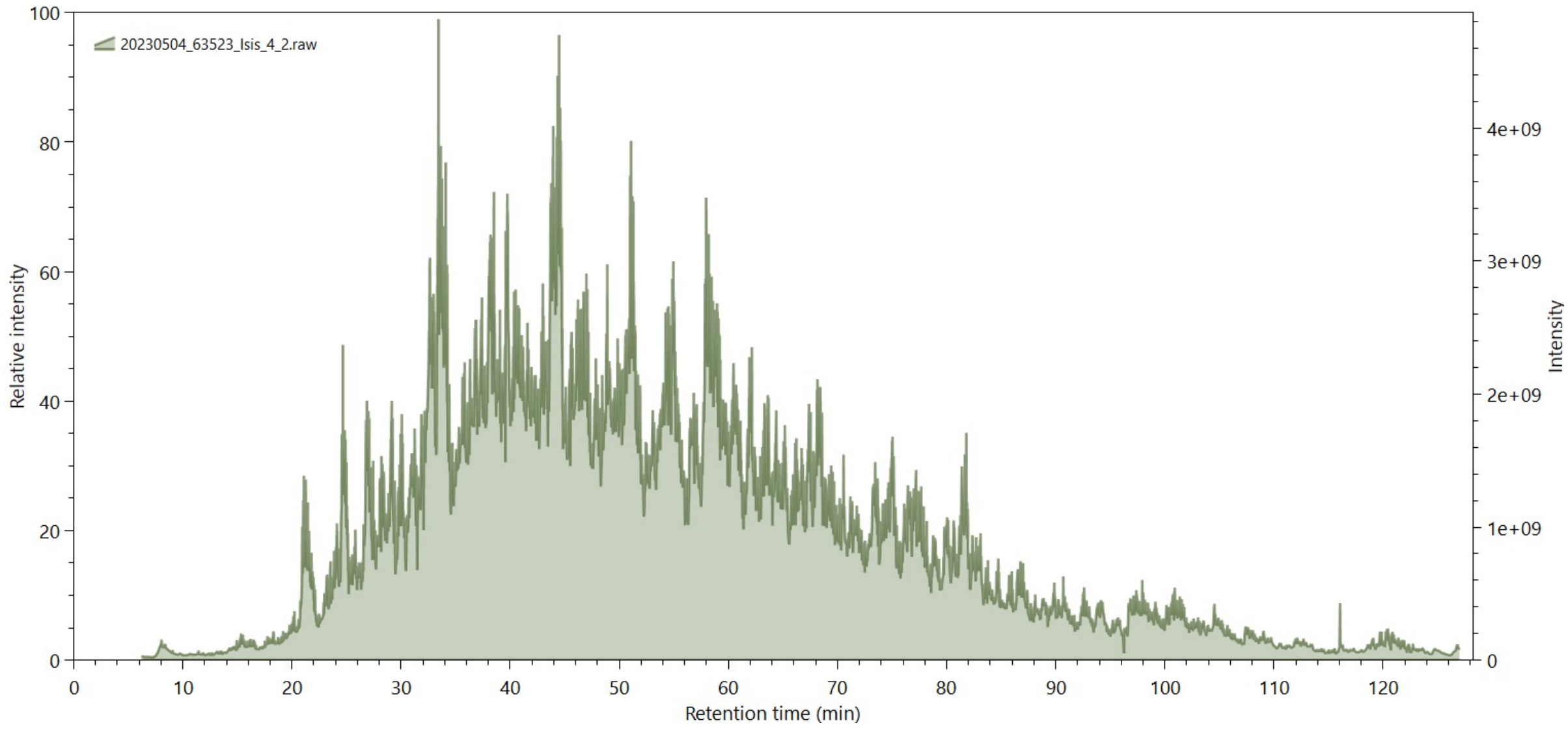
20230504_63523_Isis_4_2.raw
Relative intensity
Intensity
Retention time (min)

E

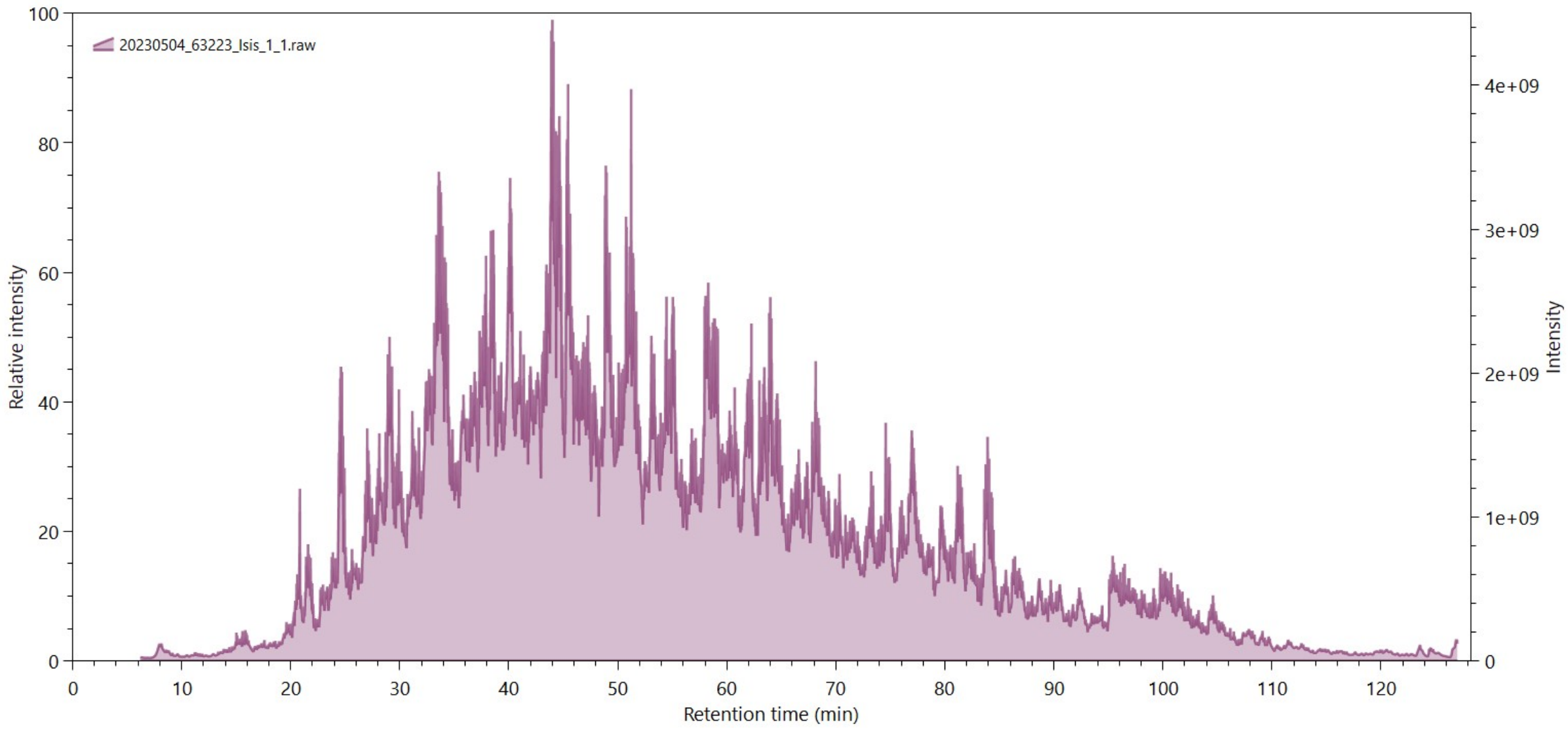
20230504_63223_Isis_1_1.raw
Relative intensity
Intensity
Retention time (min)
0
10
20
30
40
50
60
70
80
90
100
110
120
4e+09
3e+09
2e+09
1e+09

F

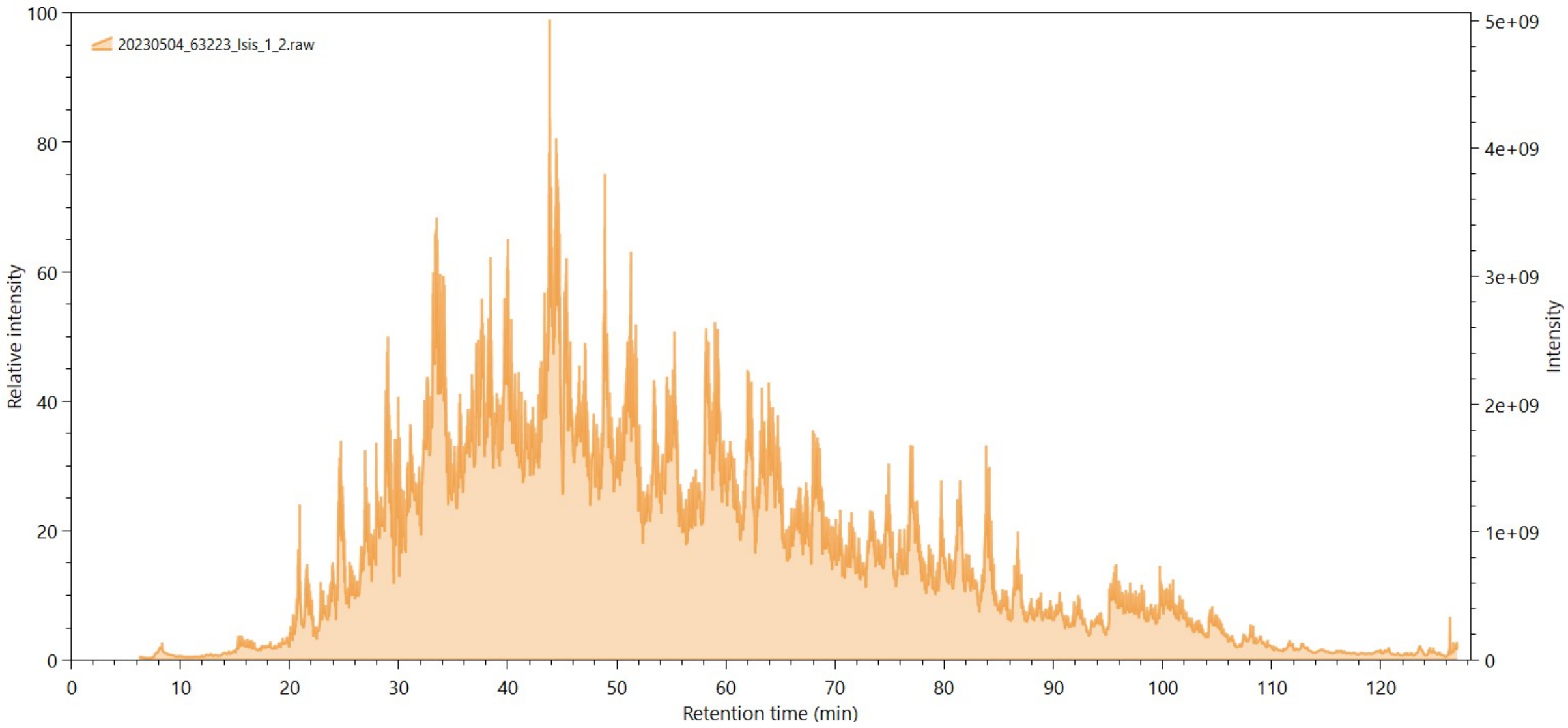
20230504_63223_Isis_1_2.raw
Relative intensity
Intensity
Retention time (min)
0
10
20
30
40
50
60
70
80
90
100
110
120
5e+09
4e+09
3e+09
2e+09
1e+09

G

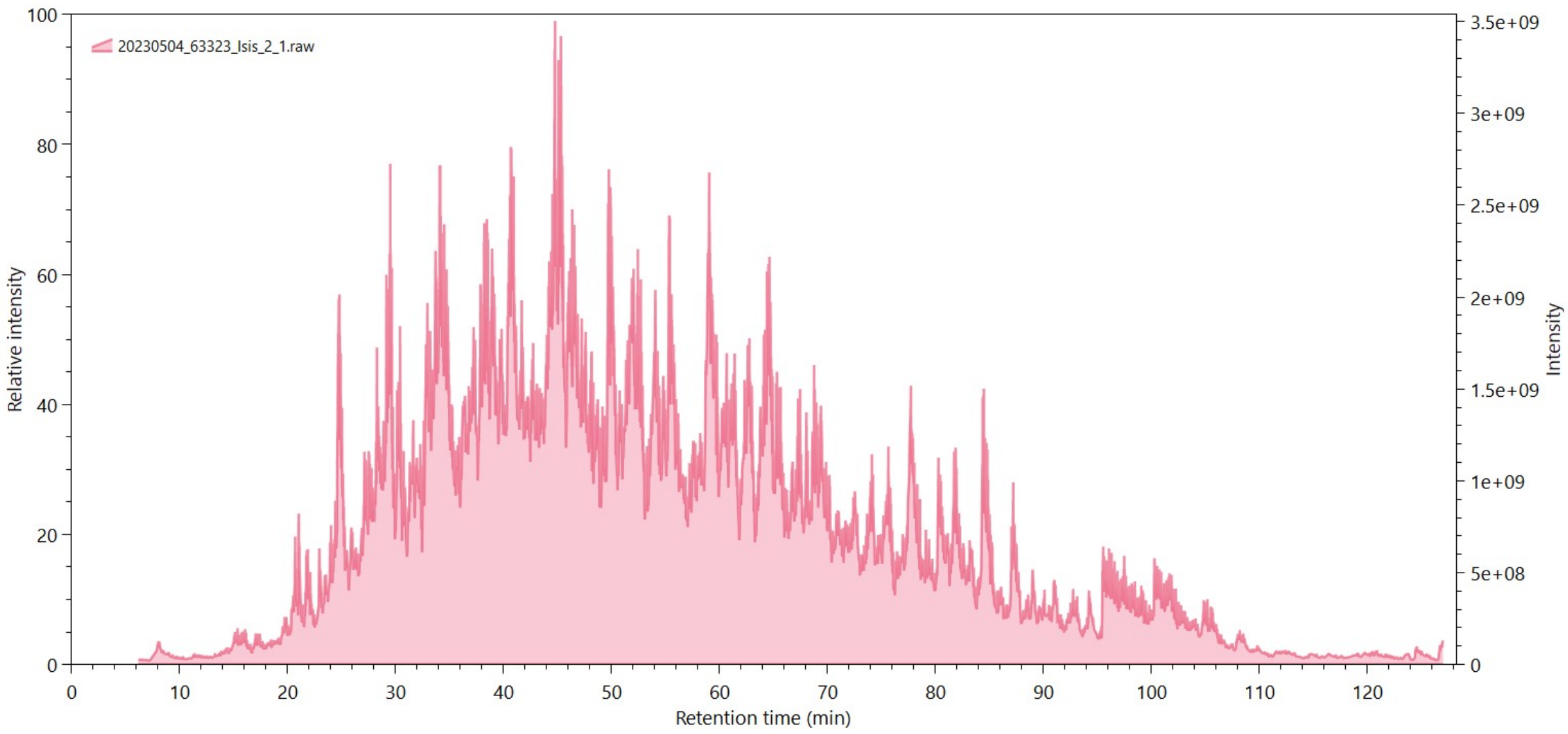

H

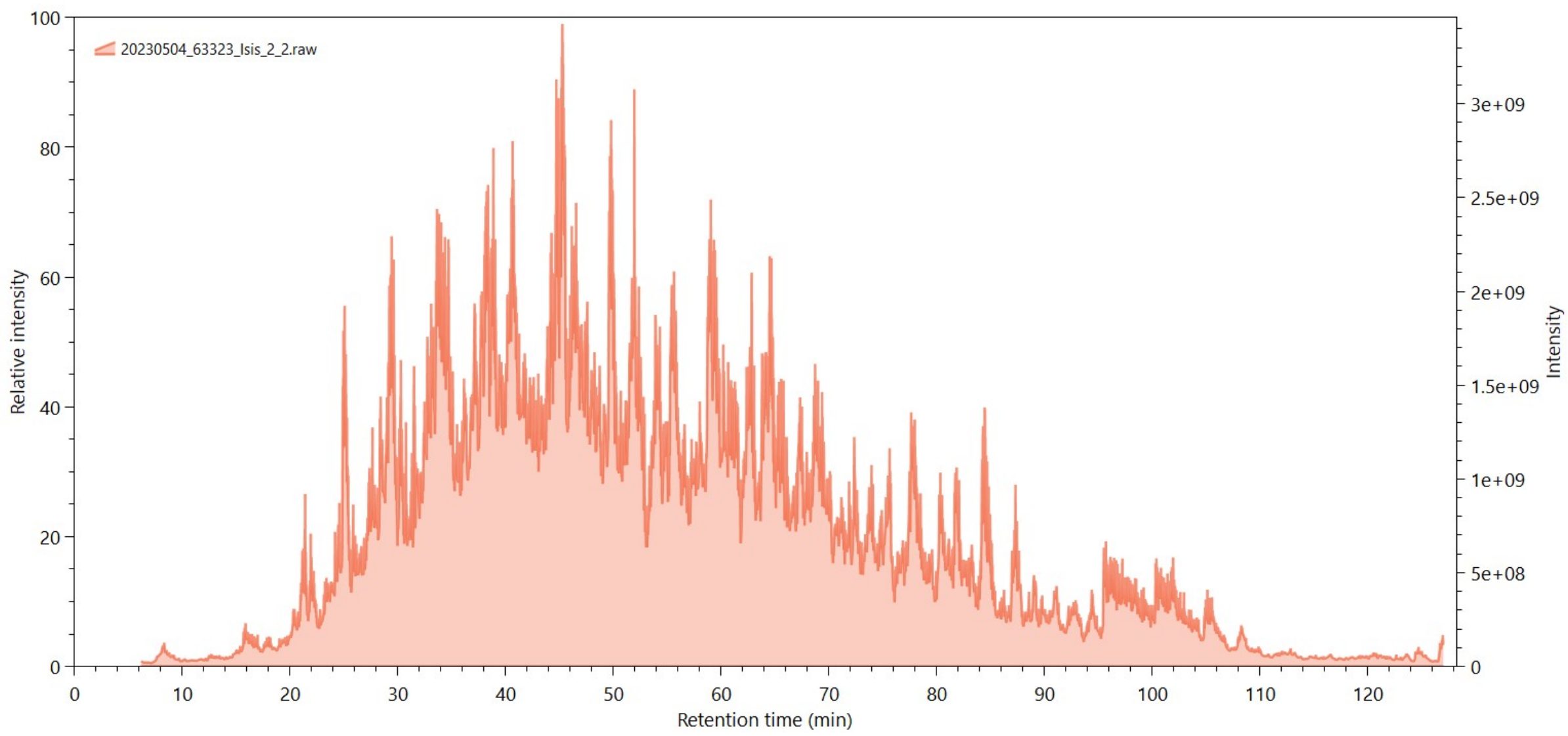

Figure S4. Chromatograms of the LC–MS/MS data acquisition of H1 and SP1C8-derived neural progenitor samples. (A) H1 neural progenitor samples, biological replica 1, technical replica 1; (B) H1 neural progenitor samples, biological replica 1, technical replica 2; (C) H1 neural progenitor samples, biological replica 2, technical replica 1; (D) H1 neural progenitor samples, biological replica 2, technical replica 2; (E) SP1C8 neural progenitor samples, biological replica 1, technical replica 1; (F) SP1C8 neural progenitor samples, biological replica 1, technical replica 2; (G) SP1C8 neural progenitor samples, biological replica 2, technical replica 1; (H) SP1C8 neural progenitor samples, biological replica 2, technical replica 2.
